# Taxonomically Informed Scoring Enhances Confidence in Natural Products Annotation

**DOI:** 10.3389/fpls.2019.01329

**Published:** 2019-10-25

**Authors:** Adriano Rutz, Miwa Dounoue-Kubo, Simon Ollivier, Jonathan Bisson, Mohsen Bagheri, Tongchai Saesong, Samad Nejad Ebrahimi, Kornkanok Ingkaninan, Jean-Luc Wolfender, Pierre-Marie Allard

**Affiliations:** ^1^Institute of Pharmaceutical Sciences of Western Switzerland (ISPSO), University of Geneva, Centre Médical Universitaire (CMU), Geneva, Switzerland; ^2^Faculty of Pharmaceutical Sciences, Tokushima Bunri University, Tokushima, Japan; ^3^Center for Natural Product Technologies, Program for Collaborative Research in the Pharmaceutical Sciences (PCRPS), University of Illinois at Chicago, Chicago, IL, United States; ^4^Department of Pharmaceutical Sciences, College of Pharmacy, University of Illinois at Chicago, Chicago, IL, United States; ^5^Department of Phytochemistry, Medicinal Plants and Drugs Research Institute, Shahid Beheshti University, G.C., Evin, Tehran, Iran; ^6^Department of Pharmaceutical Chemistry and Pharmacognosy, Faculty of Pharmaceutical Sciences and Center of Excellence for Innovation in Chemistry, Naresuan University, Phitsanulok, Thailand

**Keywords:** metabolite annotation, chemotaxonomy, scoring system, natural products, computational metabolomics, taxonomic distance, specialized metabolome

## Abstract

Mass spectrometry (MS) offers unrivalled sensitivity for the metabolite profiling of complex biological matrices encountered in natural products (NP) research. The massive and complex sets of spectral data generated by such platforms require computational approaches for their interpretation. Within such approaches, computational metabolite annotation automatically links spectral data to candidate structures *via* a score, which is usually established between the acquired data and experimental or theoretical spectral databases (DB). This process leads to various candidate structures for each MS features. However, at this stage, obtaining high annotation confidence level remains a challenge notably due to the extensive chemodiversity of specialized metabolomes. The design of a metascore is a way to capture complementary experimental attributes and improve the annotation process. Here, we show that integrating the taxonomic position of the biological source of the analyzed samples and candidate structures enhances confidence in metabolite annotation. A script is proposed to automatically input such information at various granularity levels (species, genus, and family) and complement the score obtained between experimental spectral data and output of available computational metabolite annotation tools (ISDB-DNP, MS-Finder, Sirius). In all cases, the consideration of the taxonomic distance allowed an efficient re-ranking of the candidate structures leading to a systematic enhancement of the recall and precision rates of the tools (1.5- to 7-fold increase in the F1 score). Our results clearly demonstrate the importance of considering taxonomic information in the process of specialized metabolites annotation. This requires to access structural data systematically documented with biological origin, both for new and previously reported NPs. In this respect, the establishment of an open structural DB of specialized metabolites and their associated metadata, particularly biological sources, is timely and critical for the NP research community.

## Introduction

Specialized metabolites define the chemical signature of a living organism. Plants, sponges and corals, but also microorganisms (bacteria and fungi), are known to biosynthesize a wealth of such chemicals, which can play a role as defense or communication agents (Brunetti et al., 2018). Throughout history, humans have been relying on plant derived products for a variety of purposes: housing, feeding, clothing and, especially, medication. In fact, our therapeutic arsenal is deeply dependent on the chemistry of natural products (NPs) whether they are used in mixtures, purified forms or for hemi-synthetic drug development. After a period of disregard by the pharmaceutical industry, NPs are now the object of renewed interest, partly because of the promises of the latest technological developments (Shen, 2015). Developments in metabolite profiling by mass spectrometry (MS) grant access to large volumes of high-quality spectral data from minimal amount of samples and appropriate data analysis workflows allow to efficiently mine such data (Wolfender et al., 2019). Initiatives such as the Global Natural Products Social (GNPS) molecular networking (MN) project offer both a living MS repository and the possibility to establish MN organizing MS data (Wang et al., 2016). However, despite such advancements, *metabolite identification* remains a major challenge for both NP research and metabolomics (Kind et al., 2018). Metabolite identification of a novel compound requires physical isolation of the analyte followed by complete NMR acquisition and three-dimensional structural establishment *via* X-ray diffraction or chiroptical techniques. For previously described compounds, metabolite identification implies complete matching of physicochemical properties between the analyte and a standard compound (including chiroptical properties). Metabolite identification is thus a tedious and labor-intensive process, which should ideally be reserved to novel metabolites description. Any less complete process should be defined as *metabolite annotation*. By definition, metabolite annotation can be applied at a higher throughput and offers an effective proxy for the chemical characterization of complex matrices. This process includes dereplication (the annotation of previously described molecules prior to any physical isolation process) and allows focusing isolation and metabolite identification efforts on potentially novel compounds only (Gaudêncio and Pereira, 2015).

**Figure 1:**
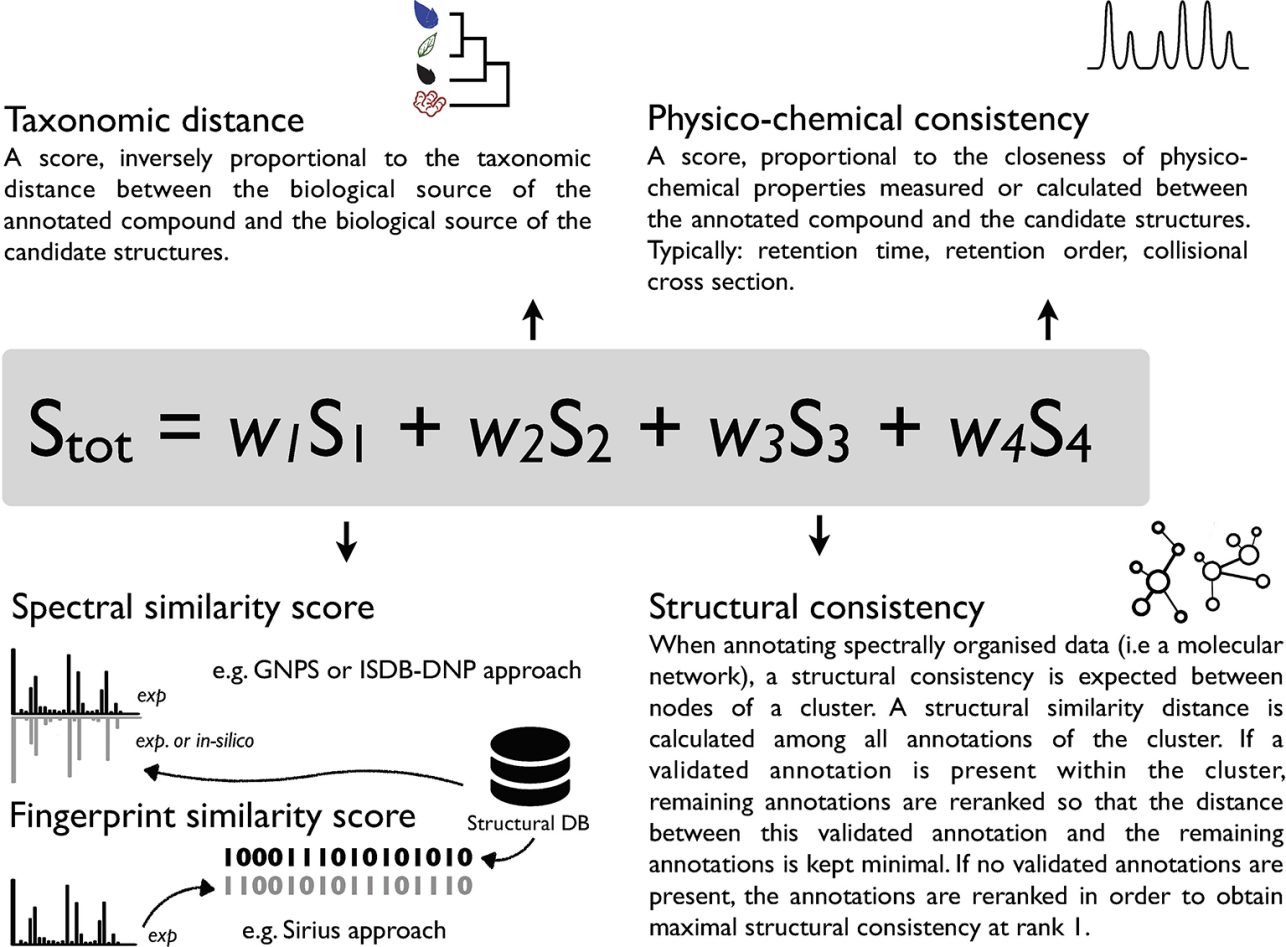
Conceptual overview of a possible metascoring system for specialized metabolite annotation incorporating 1) spectral similarity or fingerprint similarity 2) taxonomic distance between the biological source of the queried spectra and candidate annotations 3) structural consistency within a cluster [see (da Silva et al., 2018)] and 4) physico-chemical consistency (see Ruttkies et al., 2016; Bach et al., 2018). A factor (*w* *_n_*) should allow to attribute relative weight to individual scores and modulate their contribution to the overall score.

Given its sensitivity, selectivity and structural determination potential, MS is a tool of choice for metabolite annotation in complex mixtures. Various computational MS solutions have been developed to link experimental spectra to chemical structures. They can be classified into experimental rule-based strategies [MassHunter, Agilent Technologies], combinatorial fragmentation strategies [MetFrag, (Ruttkies et al., 2016)], machine learning based approaches using stochastic Markov modelling [CFM-ID, (Allen et al., 2014; Djoumbou-Feunang et al., 2019)] or predicting fragmentation trees [Sirius (Böcker et al., 2009; Dührkop et al., 2019)]. Computationally demanding ab initio calculations, modeling the gas-phase fragmentation process, have also been proposed (Bauer and Grimme, 2016). The output of such tools is, in general, a list of candidate molecules ranked according to a score. Such score can be based on a single measure (e.g. spectral similarity in CFM-annotate) (Allen et al., 2014) or integrate combined parameters (MS-Finder, Sirius) (Tsugawa et al., 2016; Dührkop et al., 2019; Tsugawa et al., 2019). The rationale behind comprehensive scoring systems is that orthogonal information (not directly related to spectral comparison) should further strengthen the metabolite annotation process. This has been illustrated in the past by using the number of literature references related to a candidate structure and basic retention time scoring based on logP in MetFrag 2.2 (Ruttkies et al., 2016). Recently, the integration of retention order prediction to an MS/MS prediction tool provided increased performance in metabolite annotation (Bach et al., 2018). Another example is the Network Annotation Propagation (NAP) approach, which takes advantage of the topology of a MN to proceed to a re-ranking of annotated candidates within a cluster where structural consistency is expected (da Silva et al., 2018). In our view, increased confidence in specialized metabolite annotation can be achieved by the establishment of a metascoring system capturing the similarity of diverse attributes shared by the queried analytes and candidate structures (Allard et al., 2017). Such metascore could for example consider 1) spectral similarity 2) taxonomic distance between the producer of the candidate compound and the annotated biological matrix 3) structural consistency within a cluster and 4) physico-chemical consistency. A conceptual overview of such metascore is illustrated in **Figure 1**. To the best of our knowledge, the automated inclusion of the taxonomic dimension within a scoring system has not been considered in current metabolite annotation strategies.

The central hypothesis of this work is directly inferred from the characteristics of the specialized metabolome. Unlike primary metabolites, which are mostly ubiquitous compounds central to organism functioning, specialized metabolites are, by definition, strongly linked to the taxonomic position of the producing organism. It thus appears desirable to consider taxonomic information when describing the chemistry of an organism. A *taxonomic filtering* process could be used to limit a database (DB) to compounds previously isolated from organisms situated within a given taxonomic distance from the biological source of the analyte to annotate. However, results of chemotaxonomic studies also highlight the presence of broadly distributed metabolites. For example, liriodenine (MUMCCPUVOAUBAN-UHFFFAOYSA-N) is a widely distributed alkaloid produced by more than 50 distinct biological sources, it is found in over 30 genera belonging to 13 botanical families. Convergent biosynthetic pathways offer intriguing example of unrelated species, shaped by evolution, that end up producing similar classes of compounds (Pichersky and Lewinsohn, 2011). To proceed to the annotation of such compounds, a *taxonomically informed scoring* allowing, both, to consider spectral similarity and taxonomic information while conserving the independence of the individual resulting scores appears as a better solution than a basic filtering process.

In the frame of this study we propose such taxonomically informed scoring system and benchmark the impact of taxonomic distance consideration on a set of 2,107 identified molecules using three different computational mass spectrometry metabolite annotation tools (ISDB-DNP, MS-Finder and Sirius).

## Results

### Conception of the Taxonomically Informed Scoring System

The constituents of specialized metabolomes, as expression products of the genome, should reflect the taxonomic position of the producing organisms. The initial hypothesis of this work is that *the attribution of a score reflecting the taxonomic distance between the biological source of the queried analyte and the one of the candidate structures, is a valuable input for a metabolite annotation process*.

**Figure 2:**
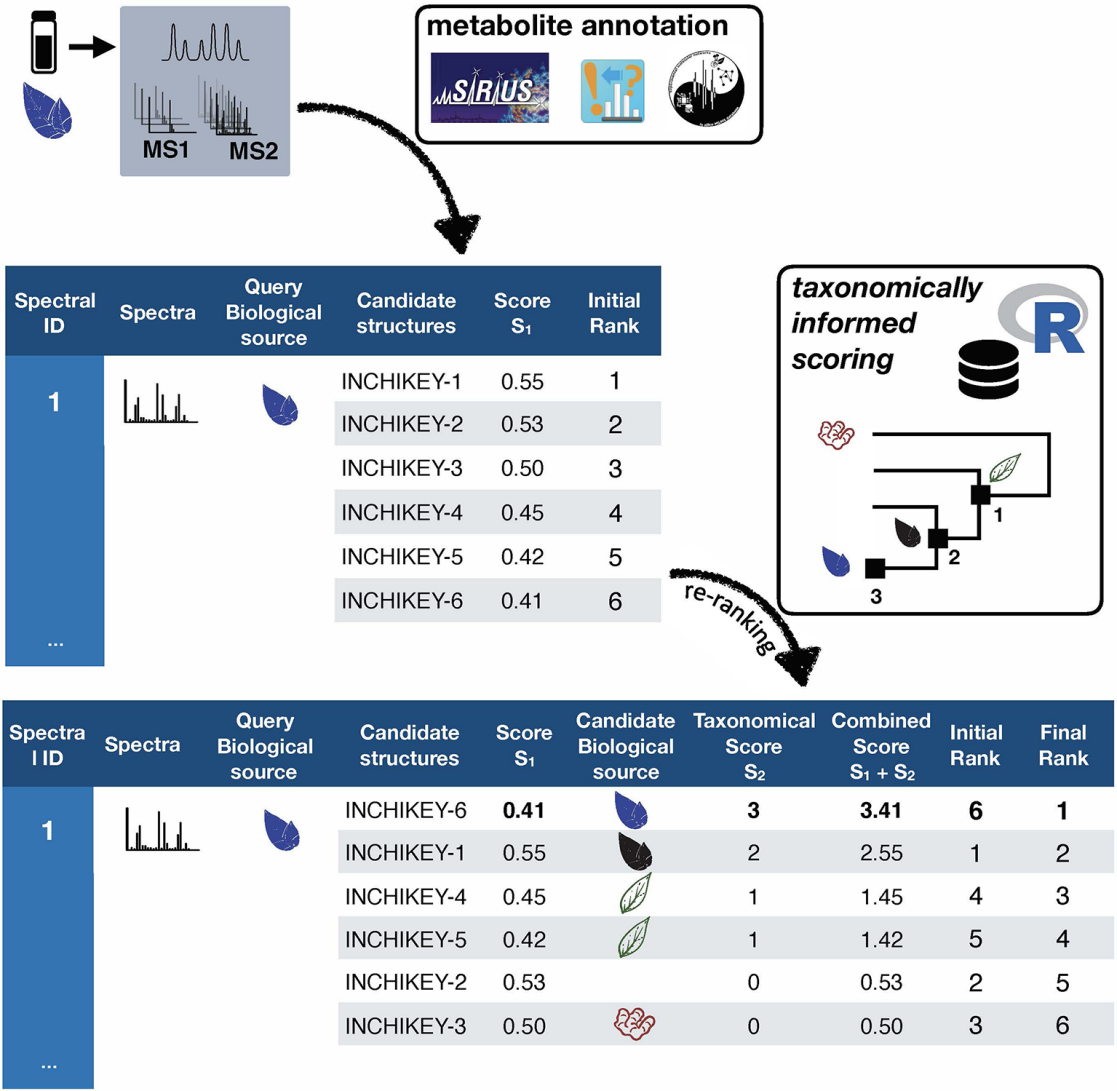
General outline of the *taxonomically informed scoring* system. Candidates’ structures are complemented with their biological sources at the family, genus and species level, when available. A score, inversely proportional to the taxonomic distance between the biological source of the standard compound and the one of the candidate compounds is given when the biological source of the candidate structures matches the biological source of the standard at the family, genus and species level, respectively. The maximum score for each candidate is then added to its spectral score to yield a complemented spectral score. Finally, candidates are re-ranked according to the complemented spectral score.

*Taxonomically informed scoring* is proposed to complement the initial score (S1 in **Figure 1**) attributed to candidate structures by existing metabolite annotation tool. To this end, the initial score is first normalized. Then, scores, inversely proportional to the taxa level difference (family < genus < species) are attributed when an exact match is observed between biological source denominations at the different taxa levels. The score corresponding to the shortest taxonomic distance is then added to the initial score. Candidates are further re-ranked according to the newly complemented score. In this study, no phylogenetic distances within taxa (e.g. family, genus or species) were considered due to high computational requirements, but the development of such an approach would be of interest. The general outline of the *taxonomically informed scoring* system is presented in **Figure 2**.

In order to apply the *taxonomically informed scoring* in a generic manner, the initial scores given by the metabolite annotation tools were rescaled to obtain values ranging from 0 (worst candidate) to 1 (best candidate). The scores, given according to the taxonomic distance between the biological source of the queried spectra and the one of the candidate compounds, were integrated in the final score by a sum. This choice allows to keep independence between individual components of the metascore (see **Figure 1**). Since the boundaries of the candidates’ normalized score in a given dataset are defined (0 to 1), the minimal score to be applied to the worst candidate for it to be ranked at the first position after *taxonomically informed scoring* is 1. Following our initial hypothesis, a score of 1 was thus given if a match between biological sources was found at the family taxa level. In the case where the initial maximal score (1) would be given to a candidate and added to a score corresponding to a match at the family level (1), a score of at least 2 should be given for a candidate having the worst score to be ranked above. A score of 2 was thus given if a match between biological sources was found at the genus taxa level. Following the same logic, a score of 3 was given for matches between biological sources at the species level.

### Benchmarking the Influence of Taxonomically Informed Scoring in Metabolite Annotation

#### Establishment of a Benchmarking Dataset

In order to establish the importance of considering taxonomic information in metabolite annotation, an experimental reference dataset constituted by molecular structures, their MS/MS spectra acquired under various experimental conditions and their biological sources, in the form of a fully resolved taxonomic hierarchy, was needed. This dataset, denominated hereafter *benchmarking dataset*, was built by combining a curated structural/biological sources dataset (obtained from the Dictionary of Natural Products (DNP)) and a curated structural/spectral dataset (obtained from GNPS librairies). Steps followed for the establishment of the benchmarking dataset are detailed below and summarized in **Supplementary Figure S3**.

##### Structural and Biological Sources Dataset

The prerequisite to apply a *taxonomically informed scoring* in a metabolite annotation process is to dispose of the biological source information of *1)* the queried MS/MS spectra and *2)* the candidate structures. To the best of our knowledge, there is currently no freely available database (DB) compiling NP structures and their biological sources down to the species level. This study uses the DNP which is commercially available and allows export of structures and biological sources as associated metadata. A curation process using the Global Names index, kept biological sources resolved against the Catalogue of Life and resulted in 219,800 entries with accepted scientific names and a full, homogeneous, taxonomy up to the kingdom level. For example, the entry initially corresponding to pulsaquinone, “Constit. of the roots of *Pulsatilla koreana*”, is converted to Plantae | Tracheophyta | Magnoliopsida | Ranunculales | Ranunculaceae | *Pulsatilla* | *Pulsatilla cernua* in the curated DB. See Material and Methods and **Supplementary Figure S3** for details concerning the curation process.

##### Structural and Spectral Dataset

The GNPS libraries agglomerate a wide and publicly available ensemble of MS/MS spectra coming from various analytical platforms and thus having different levels of quality (Wang et al., 2016). These spectral libraries were used as representative source of diverse experimental MS/MS spectra to evaluate the annotation improvement that could be obtained by applying *taxonomically informed scoring*. All GNPS libraries and publicly accessible third-party libraries were retrieved online (https://gnps.ucsd.edu/ProteoSAFe/libraries.jsp) and concatenated as a single spectral file containing 66,646 individual entries. The pretreatment described in Material and Methods, yielded a dataset of 40,138 structures (8,558 unique structures) with their experimental associated MS/MS acquired on different platforms. See **Supplementary Figure S3**.

##### Structural, Spectral and Biological Sources Dataset (Benchmarking Dataset)

To apply the *taxonomically informed scoring*, it is required that denominations of both *1)* the queried spectra and *2)* the candidate structures biological sources are resolved using a common taxonomy backbone (i.e. using the accepted denomination). It was thus necessary to build an experimental spectral dataset for which each entry had a unique structure and a properly documented biological source, which constituted the benchmarking set. The structural and spectral dataset was matched against the structural and biological sources dataset, following the procedure detailed in Material and Methods. The full processing resulted in a dataset of 2,107 individual entries (characterized NPs with no stereoisomers distinction and a unique biological source associated), which was used for the rest of this study. See **Supplementary Figure S3**.

**Figure 3:**
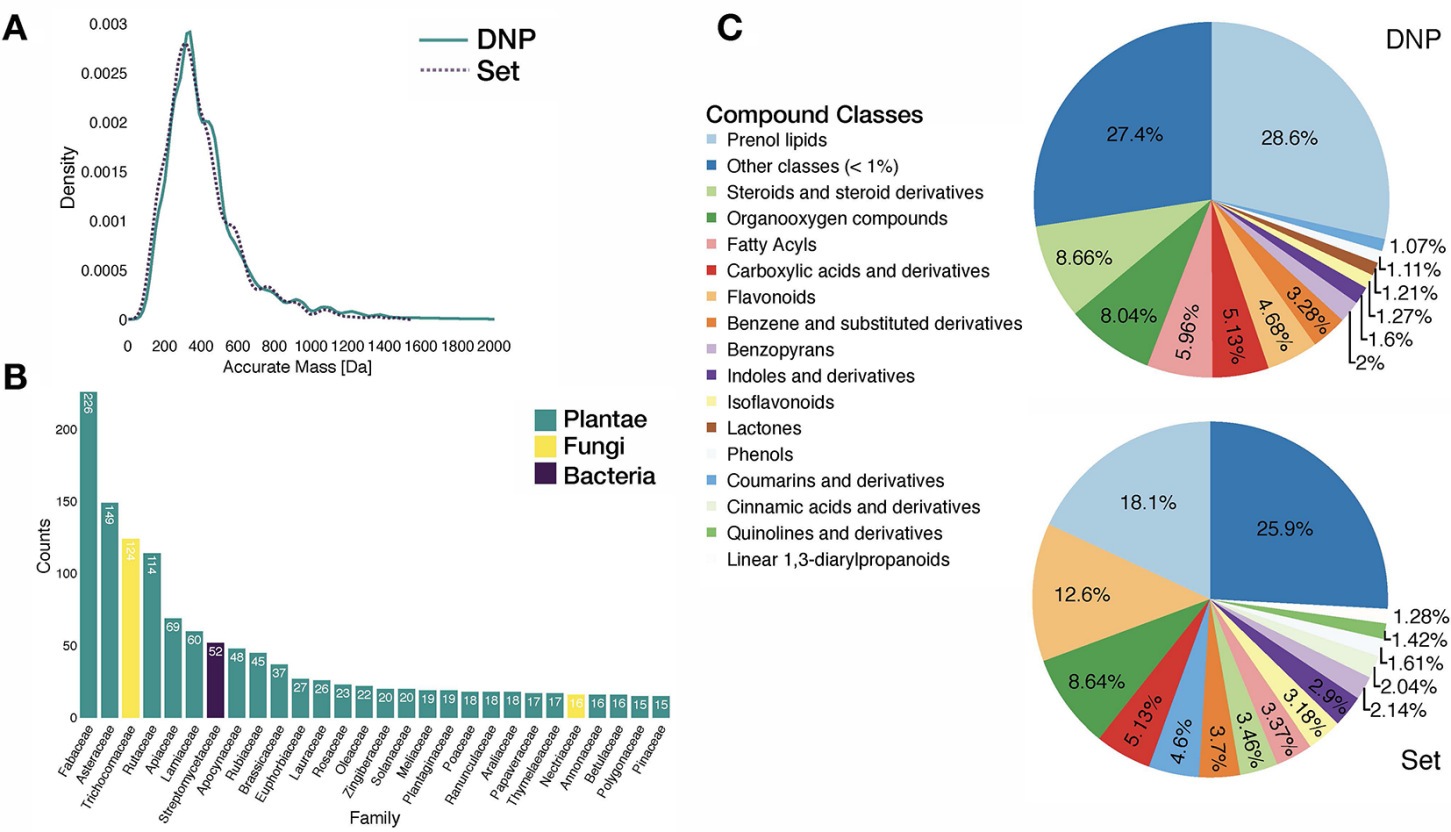
Characteristics of the benchmarking dataset. **(A)** Comparative distribution of accurate masses of entries in the DNP and in the benchmarking dataset. **(B)** Distribution of biological sources of entries in the benchmarking dataset at the family taxa level (cutoff at 15 entries per family). **(C)** Comparative distribution of chemical classes (ClassyFire Class level) within the DNP and the benchmarking set.

Analysis of the benchmarking dataset showed a chemodiversity comparable to the one of DNP (see panels **A** and **C** in **Figure 3**). Regarding the distribution of the biological sources in the benchmarking dataset, available data mostly matched plant specialized metabolites (see panel **B** in **Figure 3**). Additionally, repartition of mass analyzer types indicated the heterogeneous spectral quality of MS/MS spectra of the benchmarking dataset and was representative of commonly used analytical platforms. See repartition in **Supplementary Figure S7**.

#### Evaluation of the Improvement of Metabolite Annotation on the Benchmarking Set

In order to assess the importance of considering taxonomic information in the annotation process, the outputs of three different computational MS-based metabolite annotation solutions were considered (ISDB-DNP, MS-Finder and Sirius). The 2,107 spectra of the benchmarking dataset were queried using these tools. The precision and accuracy of structural determination with and without the use of *taxonomically informed scoring* were systematically compared according to parameters detailed in the Material and Methods section.

##### Metabolite Annotation Tools Used Isdb-Dnp

The first tool, denominated hereafter ISDB-DNP (*In Silico* DataBase—Dictionary of Natural Products) is a metabolite annotation strategy that we previously developed (Allard et al., 2016). This approach is focused on specialized metabolites annotation and is constituted by a pre-fragmented theoretical spectral DB version of the DNP. The *in silico* fragmentation was performed by CFM-ID (Allen et al., 2015). CFM-ID is, to our knowledge, the only computational solution currently available able to generate a theoretical spectrum with prediction of fragment intensity. The matching phase between experimental spectra and the theoretical DB is based on a spectral similarity measure (cosine score) performed using Tremolo (Wang and Bandeira, 2013). The scores are reported from 0 (worst candidate) to 1 (best candidate).

###### Ms-Finder

The second tool is MS-Finder. This *in silico* fragmentation approach considers multiple parameters such as bond dissociation energies, mass accuracies, fragment linkages and various hydrogen rearrangement rules at the candidate ranking phase (Tsugawa et al., 2016). The resulting scoring system range from 1 (worst candidate) to 10 (best candidate).

###### Sirius

The third tool to be used is Sirius 4.0. It is considered as a state-of-the-art metabolite annotation solution, which combines molecular formula calculation and the prediction of a molecular fingerprint of a query from its fragmentation tree and spectrum (Dührkop et al., 2019). Sirius uses a DB of 73,444,774 unique structures for its annotations. The resulting score is a probabilistic measure ranging between negative infinity (worst candidate) and 0 (best candidate).

##### Computation of the Taxonomically Informed Score

R scripts were written to perform *1)* cleaning and standardization of the outputs, *2) taxonomically informed scoring* and re-ranking. First, the outputs were standardized to a table containing on each row: the unique spectral identifier (CCCMSLIB N°) of the queried spectra, the short InChIKey of the candidate structures, the score of the candidates (within the scoring system of the used metabolite annotation tool), the biological source of the standard compound and the biological source of the candidate structures. As described above, a score, inversely proportional to the taxonomic distance between the biological source of the annotated compound and the biological source of the candidate structure, was given when both matched at the family (score of 1), genus (score of 2) or species level(s) (score of 3). A sum of this score (1 to 3) and the original score (0 to 1) yielded the *taxonomically informed score*. This *taxonomically informed score* was then used to re-rank the candidates from highest to lowest score.

##### Results Before Taxonomically Informed Scoring

**Figure 4:**
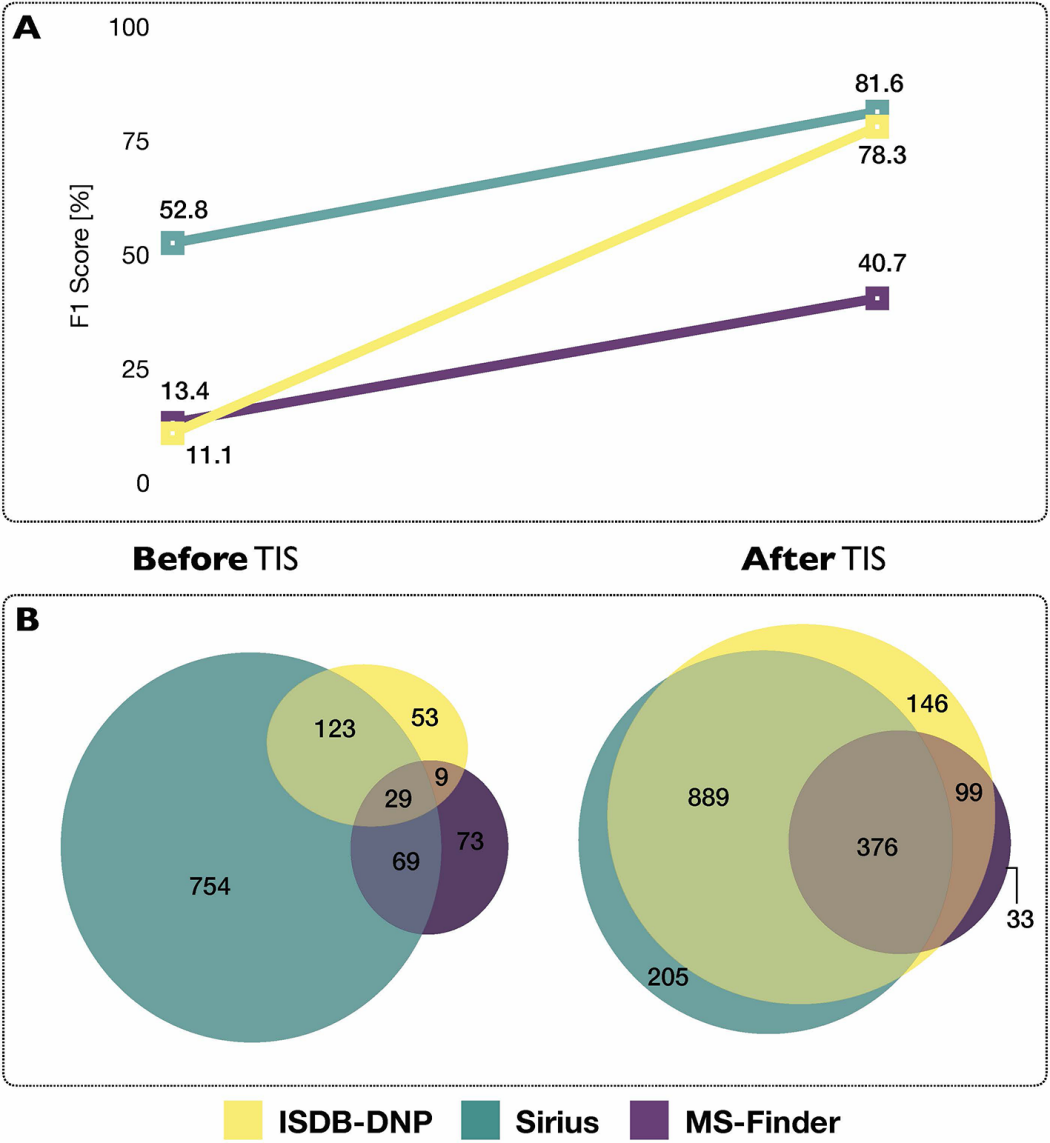
**(A)** Influence of the *taxonomically informed scoring* (TIS) on the F1 score of each metabolite annotation tool. The F1 score offers a global evaluation of the precision and recall rate of the annotation process, the higher the better. **(B)** Venn diagrams representing common and unique correct annotations of each tool at rank 1 before and after the *taxonomically informed scoring* step.

Using each tools’ initial scoring system, on the 2,107 experimental MS/MS spectra constituting the benchmarking set, the ISDB-DNP returned 214 (10.2%) correct annotations at rank 1, Sirius 975 (46.3%) and MS-Finder 180 (8.5%). The total number of unique correct annotations ranked first covered by ISDB-DNP, Sirius and MS-Finder prior to *taxonomically informed scoring* reached 1110 or 52.7% of the benchmarked dataset. Out of these, 29 (less than 1.4%), were common to all three tools, indicating the interest of considering various annotation tools when proceeding to metabolite annotation. Venn diagram in **Figure 4** illustrates the complementarity of returned annotations. Within all candidates (all ranks), the ISDB-DNP returned 1,750 correct annotations, Sirius 1,589 and MS-Finder 574. The ROC curves outline the number of correct hits outside first rank and indicate remaining improvement potential. See **Supplementary Figure S4**.

##### Results After Taxonomically Informed Scoring

After *taxonomically informed scoring* and reranking, the number of correct annotations at rank 1 increased to 1,510, 1,508 and 546, respectively for ISDB-DNP, Sirius and MS-Finder. The total number of correct annotations covered by all ISDB-DNP, Sirius and MS-Finder after *taxonomically informed scoring* reached 1786 or 84.8% of the benchmarked dataset. Interestingly, more than 10-fold increase after *taxonomically informed scoring* was also observed for the correctly annotated metabolite commonly returned by the three tools 376 (17%). It has to be noted that no stereoisomer distinction could be performed since all correct matches were assessed based on short InChIKey comparison.

F1 score (harmonic mean of precision and recall rate) was used in order to evaluate the impact of the *taxonomically informed scoring*. More details on the establishment of the score can be found in Material and Methods. The F1 scores of the three metabolite annotation tools before and after *taxonomically informed scoring* are displayed on **Figure 4**. The *taxonomically informed scoring* stage led to a systematic increase of the F1 score for the benchmarked tools. This increase was 7-fold (ISDB-DNP), 1.5-fold (Sirius) and 3-fold (MS-Finder).

### Optimization of Scores Combination for the Taxonomically Informed Scoring

In order to verify our initial hypothesis and define the optimal scores combination (at the family, genus and species taxa level) to be applied for *taxonomically informed scoring* we proceeded to an optimization of the *taxonomically informed scoring* function.

**Figure 5:**
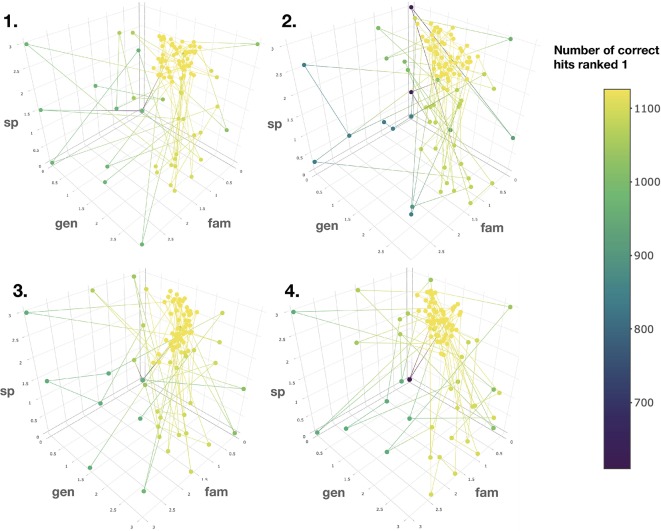
Results of the Bayesian optimization converge toward the optimal scores combination required for a maximal number of correct annotations ranked at the first position. This is observed for four randomly degraded training sets (first optimization round displayed). sp, gen and fam axes correspond to the score given when a match is found at the species, genus or family taxa level, respectively. Results confirm that the applied scores should be *inversely proportional to the taxonomic distance between the biological source associated with the queried spectra and the biological source of the candidate structures.*

To this end, the taxonomic information related to candidate annotations was artificially degraded. This step allowed to mimic a “real life” case in which candidate annotation’s taxonomic metadata are not necessarily complete or correct down to the species level. Using the procedure detailed in the corresponding Material and Methods section, a Bayesian optimization algorithm was applied four times on four randomized datasets. It quickly converged (100 iterations) towards a global maximum (max 1,126 hits, see **Figure 5**). The optimal scores were found to be 0.81, 1.62 and 2.55 for the family, genus and species taxa level, respectively. Such scores are dependent on the nature and completeness of the employed taxonomic metadata. However, the results obtained when applying the Bayesian optimization on the annotation sets for which taxonomic metadata was randomly degraded, indicated that optimal results were systematically obtained when *the attributed scores were inversely proportional to the taxa hierarchical position*, thus confirming our initial hypothesis.

### Application of the Taxonomically Informed Scoring to the Annotation of Metabolites From *Glaucium* Sp

The interest of the *taxonomically informed scoring* was further illustrated for the annotation of specialized metabolites from *Glaucium* species (Papaveraceae family). Three species, *G. grandiflorum*, *G. fimbrilligerum* and *G. corniculatum* were studied. The ethyl acetate and methanolic extracts of the three species were profiled by UHPLC-HRMS in positive ionization mode using a data-dependent MS/MS acquisition. After appropriate data treatment and molecular network generation (see corresponding Material and Methods section), the *taxonomically informed scoring* was used to re-rank the candidate annotation returned by the ISDB-DNP. Best five hits were kept. We especially focused on the two major compounds (MS signal intensity) of *G. grandiflorum*. These were feature *m/z* 342.1670 at 1.42 min and *m/z* 356.1860 at 1.83 min. According to the optimization results (see previous section), a score of 0.81 was given to candidates for which the biological source was found to be Papaveraceae at the family level, 1.62 to *Glaucium* at the genus level and 2.55 to *G. grandiflorum* at the species level. The results of the *taxonomically informed scoring* annotation for feature *m/z* 342.1670 at 1.42 min are presented in **Table 1**. See **Supplementary Table S5** for annotation results concerning feature *m/z* 356.1860 at 1.83 min.

**Table 1:** Output of the taxonomically informed scoring annotation using ISDB-DNP for feature m/z 342.1670 at 1.42 min. Predicentrine, the correct annotation, which was initially ranked at the 9th position, is ranked at the first position after taxonomically informed scoring.

ClusterID	Structure	Short IK	Molecule Name	Family	Genus	Species	Family Score	Genus Score	Species Score	Max Taxo Score	SpectralScore	Normalize Spectral Score	Combined Spectral + Taxo Score	Rank Initial	Rank Final
1772	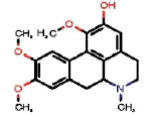	OUTYMWDDJORZOH	Predicentrine	Papaveraceae	Glaucium	Glaucium oxylobum	0.81	1.62	0	1.62	0.36	0.23	1.85	9	1
1772	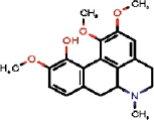	QELDJEKNFOQJOY	Isocorydine	Papaveraceae	Glaucium	NA	0.81	1.62	0	1.62	0.34	0.22	1.84	11	2
1772	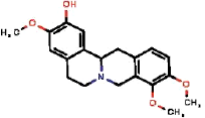	KDFKJOFJHSVROC	Isocorypalmine	Papaveraceae	Glaucium	Glaucium fimbrilligerum	0.81	1.62	0	1.62	0.3	0.14	1.76	28	3
1772	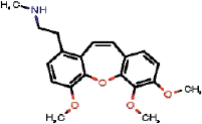	JADHMUPTWPBTMT	Secosarcocapnidine Me ether, N-De-Me	Papaveraceae	Sarcocapnos	Sarcocapnos crassifolia	0.81	0	0	0.81	0.4	0.32	1.13	1	4
1772	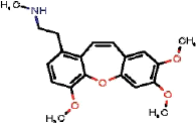	WNBUTZHPPULVTP	Secocularidine Me ether, N-de-Me	Papaveraceae	Ceratocapnos	Ceratocapnos claviculata	0.81	0	0	0.81	0.39	0.29	1.1	2	5

Both features were targeted within the extract and, after isolation, the structure of their corresponding compound was determined by 1D and 2D NMR measurements (see spectra in **Supplementary Material S1** and **S2**). NMR spectra of feature *m/z* 342.1670 at 1.42 min matched to the literature reported spectra for predicentrine (Guinaudeau et al., 1979). NMR Spectra of feature *m/z* 356.1860 at 1.83 min matched to glaucine (Huang et al., 2004). In both cases, the candidate structure proposed *via* the *taxonomically informed scoring* annotation at rank 1 was found to be correct. With the classical spectral matching process, the correct candidates were initially ranked at positions 9 and 7 for predicentrine and glaucine, respectively (see **Table 1** and **S5** in **Supplementary Material**).

Additional predicentrine analogues were annotated in the corresponding cluster (see examples in **S6** in **Supplementary Material**).

## Discussion

The metabolite annotation process can be boiled down to the comparison of attributes (e.g. exact mass, molecular formula (MF) fragmentation spectra) of the queried analyte to attributes of candidate structures present in a DB. When HRMS and appropriate heuristic filters are used, the establishment of the MF of the analyte is relatively straightforward (Kind and Fiehn, 2007). However, this is not sufficient to proceed to metabolite annotation given the isomeric nature of numerous NP: over all compounds reported in the DNP, less than 10% have a unique chemical formula, the average number of compounds per molecular formula is 8.6 and a maximum of 1,274 isomers is found for C_15_H_20_O_4_. With an MS1 analysis relying on exact mass only, no ranking between those isomers is possible. Computational metabolite annotation tools allow to attribute a score to candidate structures and, thus, to discriminate isomeric molecules. However, MF and fragmentation spectra are not the only attributes which can be compared in the metabolite annotation process. Specialized metabolites, as products of biosynthetic clusters themselves part of the genome, are tightly linked to the taxonomic position of the producing organisms (Hoffmann et al., 2018; Ernst et al., 2019). Here, we demonstrate that the taxonomic distance between the biological source of the queried compound and the biological source of the candidate structures is a valuable attribute to integrate into the metabolite annotation process. We show that such information can be considered in a *taxonomically informed scoring* system and automatically applied to the outputs of different computational metabolite annotation programs. The consideration of taxonomic information was shown to systematically improve the F1 score of the evaluated solutions (ISDB-DNP, Sirius, MS-Finder) with a 1.5 to 7-fold increase. The advantage of considering such information in the metabolite annotation process are thus observed *independently* of the tools and their associated structural DBs.

It is worth noting that this benchmarking was carried to evaluate the importance of considering taxonomic information during the metabolite annotation process. *It was not meant to compare the performances of the tools*. Indeed, all compounds of the benchmarking dataset are present in the DNP, and the ISDB-DNP tool, which is by definition backed by the same DB is thus favored. On the other hand, the GNPS spectral libraries were also part of the Sirius training set. Furthermore filters for the selection of [M+H]^+^ adducts and for the filtering MS/MS spectra (500 most intense peaks) were applied to meet restriction of the ISDB-DNP and MS-Finder, respectively. Finally, a number of entries (197) of the benchmarking dataset were found to have large mass difference (> 0.01 Da) between their experimental parent ion mass and their calculated exact mass. For example, cevadine [M+H]^+^ (CCMSLIB00004689734) had an experimental parent ion mass of 632.386 Da, while its calculated exact mass is 591.3407 Da (C_32_H_49_NO_9_). Of course, such erroneous entries cannot be identified by the computational metabolite annotation tools (the list of these problematic entries is available online Problematic_entries.csv). Altogether these elements prevent a fair comparison of each tool’s performances. Another precautionary statement concerns the results of the optimization on candidate datasets for which taxonomic information had been randomly degraded at multiple taxa level. This optimization indicated, for the ISDB-DNP results, that the optimal combination of scores was 0.81, 1.62 and 2.55 (family, genus and species taxa level, respectively). Such results should be taken with caution, and not as absolute optimal values, as such optimization process are heavily dependent on the training sets. Nevertheless, the optimization indicates that the best results were repeatedly obtained when the assigned scores were inversely proportional to the taxonomic distance between the biological sources of, both, the queried spectra and the candidate structures.

Other limitations of the described metabolite annotation strategy include its application range and prerequisites. Indeed, it is important to note that such *taxonomically informed scoring* system will mostly benefit the annotation process of *specialized metabolites* and not ubiquitous molecules (e.g. coming from the primary metabolism) for obvious reasons. Furthermore, it heavily depends on the availability and quality of DBs compiling structures and their biological sources reported as a fully and homogeneously resolved taxonomy. To the best of our knowledge, such DBs are not publicly available and downloadable at the moment. KNApSAcK (http://www.knapsackfamily.com/KNApSAcK/) is a comprehensive species-metabolite relationship database compiling 116,315 metabolite-species pairs entries, it is accessible online but not downloadable. Other databases such as FooDB (http://foodb.ca) are fully downloadable but however focused on food-related metabolites, furthermore the biological sources field is not standardized. The NPAtlas (https://www.npatlas.org/) is an interesting initiative, however biological sources information down to the species level is only accessible in query mode and the DB is limited to 24,594 metabolites of microbial origin only. The Dictionary of Natural Products, which we used in this study is the widest compilation of structure/biological sources pairs, but is only available commercially. Furthermore, the biological sources are reported as a free text field (codes are available only for the family taxa levels and above), thus requiring tedious standardization and name resolving.

It is therefore important for the community to start the systematic reporting of biological sources, together with spectral and structural information, when documenting novel metabolites. In fact, reporting newly described biological occurrence should be encouraged even for previously described metabolites. However, the policy of most journals in NP research is to accept for publication only description of novel and bioactive structures, which hinders these potentially informative reports. The GNPS spectral libraries (https://gnps.ucsd.edu/ProteoSAFe/libraries.jsp) and MassIVE repositories (https://massive.ucsd.edu/ProteoSAFe/static/massive.jsp) appear as optimal places, at the moment, to compile and share NP spectral and structural information. However, although free text comments can complement the documentation of an entry, no standardized fields are available to report the biological sources of the uploaded spectra. The creation of such a feature, ideally directly linking the entered biological sources to existing taxonomy backbones such as GBIF (https://www.gbif.org) or Catalogue of Life (http://www.catalogueoflife.org), would be extremely useful. A recent initiative, the Pharmacognosy Ontology (PHO), that builds on 50 years of development of NAPRALERT (https://www.napralert.org) is aimed at providing a Free and Open resource that will link taxonomical, chemical and biological data (http://ceur-ws.org/Vol-1747/IP12_ICBO2016.pdf). Of course, and in addition to correct and systematic biological sources occurrence reporting in dedicated DBs, it is of utmost importance to count on the expert knowledge of trained taxonomists specialized in the classification of living organisms. But it seems that today, unfortunately, these people are very few (Ajmal Ali and Choudhary, 2011; Drew, 2011).

Building on the proposed *taxonomically informed scoring*, further developments will pass by the consideration of more accurate quantification of taxonomic distances and by strengthening the metascoring system. Indeed, the approach presented here only considers the identity between the biological sources, at different taxa level, of the query compounds and the ones of the candidate structures. Taking into account a more precise phylogenetic position within or across taxa, for example *via* the calculation of taxonomic distinctiveness indexes (Clarke and Warwick, 1998; Weikard et al., 2006), could offer a more accurate distance and eventually improve such *taxonomically informed scoring* process. Such calculations could however reveal to be computationally demanding to realize on the fly. On another plan, efforts remain to be done towards the establishment of a global metascore (see **Figure 1**). Integrating the proposed taxonomic distance scoring (S_2_ in **Figure 1**) with the maximal number of available metadata (S_3_, S_4_, …) when proceeding to metabolite annotation should only be beneficial to such process. However, problematics such as the individual weights (*w*_1_, *w*_2_, *w*_3_,…) to attribute to each individual score of the metascore will have to be addressed.

## Conclusion

Efficient characterization of specialized metabolomes is a key challenge in metabolomics and NP research. Recent technical advances allow access to an ever-increasing amount of data, raising the need for *ad hoc* computational solutions for their interpretation. The metabolite annotation process, which can be resumed to the comparison of attributes of the queried features against attributes of the candidate structures can benefit from information complementary to the classically used MS/MS fragmentation. Ideally, the quantification of multiple attributes’ similarities (or dissimilarities) should be integrated within a metascoring system. Here, we demonstrate that the consideration of the taxonomic distance separating the biological sources of both the queried analytes and the candidate structures can drastically improve the efficiency of existing MS-based computational metabolite annotation solutions. Metabolite annotation is crucial to guide chemical ecology research or drug discovery projects. More than two hundred years later, the present work thus supports the first of De Candolle’s assumptions, *“Plant taxonomy would be the most useful guide to man in his search for new industrial and medicinal plants”* (de Candolle, 1804). His correlated postulate, *“Chemical characteristics of plants will be most valuable to plant taxonomy in the future”*, will be equally interesting to verify with computational approaches. Various strategies have been proposed to exploit structural (or biosynthetic) relationships among metabolites and further organize the producing organisms (Liu et al., 2017; Junker, 2018; Ernst et al., 2019; Kang et al., 2019) and interesting developments will appear once robust metabolite annotation solution are coupled to comprehensive DBs compiling structures and their biological sources. Indeed, specialized metabolome annotation could be a novel way to infer the taxonomic position of an unknown sample, just as valid as a genetic sequencing. Metabolite annotation can benefit from taxonomy and taxonomic relationships can be inferred from precise metabolite characterization. Efforts in both directions should thus fuel a *virtuous cycle of research aiming to better understand Life and its chemistry*.

## Material and Methods

### Outline and Implementation of the Taxonomically Informed Scoring System

To evaluate the importance of considering taxonomic information in the annotation process, three different computational mass spectrometry-based metabolite annotation tools were used (namely, ISDB-DNP, MS-Finder and Sirius). This resulted in three different outputs constituted by a list of candidates returned by each tool for the entries of the benchmarking dataset. These candidates were ranked according to the scoring system of each tool. R scripts in the form of markdown notebooks were written to perform *1)* cleaning and standardization of the outputs (1_taxo_cleaner.Rmd) *2) taxonomically informed scoring* and re-ranking (2_taxo_scorer.Rmd). First, the outputs were standardized to a table containing on each row: a unique spectral identifier (CCMSLIB N°) of the queried spectra, the short InChIKey of the candidate structures, the score of the candidates (within the scoring system of the used metabolite annotation tool), the biological source of the standard compound and the biological source of the candidate structures. As described in the results section, a score, inversely proportional to the taxonomic distance between the biological source of the annotated compound and the one biological source of the candidate structure, was given when an exact match was found between both biological sources at the family, genus or/and species level(s). A sum of this score and the original score yielded the *taxonomically informed score*. This score was then used to re-rank the candidates. See **Figure 2** for a schematic overview of the *taxonomically informed scoring* process. Scripts are available at https://github.com/oolonek/taxo_scorer. 

### Dataset Preparation

#### Structural and Biological Sources Dataset

In the Dictionary of Natural Products (v 27.1), taxonomic information appears in two fields. The *Biological Source* field, which is constituted by a free text field reporting occurrence of a specific compound and the *Compound Type* field which reports various codes corresponding to molecule classes or taxonomic position at the family level. As an example, for the entry corresponding to larictrin 3-glucoside (ODXINVOINFDDDD-UHFFFAOYSA-N), the Biological Source field indicates “Isol. from *Larix* spp., *Cedrus* sp. and other plant spp. Constit. of *Vitis vinifera* cv. Petit Verdot grapes and *Abies amabilis*.” and the Compound Type field indicates “V.K.52600 W.I.40000 W.I.35000 Z.N.50000 Z.Q.71600” suggesting that biological sources are found in the Phyllocladaceae (Z.N.50000) and Vitaceae family (Z.Q.71600). The biological source information is reported in a non-homogeneous way and multiple biological sources are reported in the same row. In order to extract taxonomic information out of the free text contents, the *gnfinder* program (https://github.com/gnames/gnfinder) was used. Gnfinder takes UTF8-encoded text as inputs and returns back JSON-formatted output that contains detected scientific names. It automatically detects the language of the text and uses complementary heuristic and natural language processing algorithms to detect patterns corresponding to scientific binomial or uninomial denomination. Gnfinder was forced for English language detection. In addition to scientific denomination extraction, gnfinder allows to match the detected names against the Global Names index services (https://index.globalnames.org). The preferred taxonomy backbone was set to be Catalogue of Life. This last step allowed to return the full taxonomy down to the entered taxa level. It also allows to resolve synonymy. Since gnfinder is designed to mine raw texts, the JSON formatted output indicates the position of the detected name in the original input by character position. A python script was written to output a .csv file with the found name and taxonomy in front of the corresponding input. When multiple biosources were found for an entry, this one was duplicated in order to obtain a unique structure/biological source pair per row. The script is available online (gnfinder_field_scrapper.py).

#### Structural and Spectral Dataset

All GNPS libraries and publicly accessible third-party libraries were retrieved online (https://gnps.ucsd.edu/ProteoSAFe/libraries.jsp) and concatenated as a single spectral file (Full_GNPS_lib.mgf) in the .mgf format. A python Jupyter notebook (mgf_filterer.ipynb) was created to filter.mgf spectral file according to specific parameters: maximum and minimum number of fragments per spectrum and defined spectral ID (e.g. CCMLIB N°). The spectral file was filtered to retain only entries having at least 6 fragments. For spectra containing more than 500 fragments, only the 500 most intense were kept. A second python Jupyter notebook (GNPS_parser_cleaner.ipynb) was written to proceed to *1)* extraction of relevant metadata (parent ion mass, SMILES, InChI, library origin, source instrument, molecule name and individual spectrum id value (CCMSLIB N°) *2)* filtering entries having at least one structural information associated (SMILES and/or InChI) and corresponding to protonated adducts and *3)* converting structures to their InChIKey, a 27-character hashed version of the full InChI. The InChIKey conversion was realized using the RDKit 2019.03.1 framework (RDKit: Open-source cheminformatics; http://www.rdkit.org). This resulted in a structural dataset (GNPS_lib_structural.tsv) of 40138 entries constituted by 8558 unique compounds. The dataset was further filtered to keep entries which parent masses were comprised between 100 and 1,500 Da. Duplicate structures and stereoisomers were removed by keeping distinct InChIKey according to the first layer (first 14 characters) of the hash code. This spectral dataset encompasses spectra acquired on a variety of MS platforms (See **Supplementary Figure S7**). Scripts are available at https://github.com/oolonek/taxo_scorer. Input and output data are available on OSF at the following address (https://osf.io/bvs6x/). 

#### Structural, Spectral, and Biological Sources Dataset (Benchmarking Dataset)

Once the structural and biological sources dataset and the structural and spectral datasets were prepared (as described above), both were joined in order to attribute a biological source to each spectrum. The scripts used to proceed to the merging step are part of the python Jupyter notebook (GNPS_parser_cleaner.ipynb). Since in most cases it is not expected to differentiate stereoisomers based on their MS spectra, the combination of both datasets was made using the short InChIKey (first 14 characters of the InChIKey) as a common key. In this merging process, only entries having biological source information resolved against the Catalogue of Life and complete down to the species level were retained. However, this merging implied that, for a given biological source, the information on the 3D aspects of the structure was lost. While this was not an issue for the benchmarking objective of this work the resulting dataset does not constitute a reliable occurrence dataset for annotation that needs stereoisomers to be differentiated. The resulting dataset containing structural, spectral and biological sources information was constituted by 2107 distinct entries and was named *benchmarking dataset*. The scripts allowing to generate the benchmarking dataset are available at https://github.com/oolonek/taxo_scorer. The benchmarking dataset spectral data (benchmarking_dataset_spectral.mgf), and associated metadata (benchmarking_dataset_metadata.tsv) are available at the following address (https://osf.io/bvs6x/). 

### Computational Metabolite Annotation Tools

#### ISDB-DNP

The ISDB-DNP (*In Silico* DataBase—Dictionary of Natural Products) is a metabolite annotation workflow that we previously developed (Allard et al., 2016). A version using the freely available Universal Natural Products Database (ISDB-UNPD) is available online (http://oolonek.github.io/ISDB/). This approach is focused on specialized metabolites annotation and is constituted by a pre-fragmented theoretical spectral DB version of the DNP. The *in silico* fragmentation was performed using CFM-ID, a software using a probabilistic generative model for the fragmentation process, and a machine learning approach for learning parameters for this model from MS/MS data (Allen et al., 2015). CFM, is, to the best of our knowledge, the only solution available at the moment allowing to output a spectrum with fragment intensity prediction. The matching phase between experimental spectra and the theoretical DB is based on a spectral similarity computation performed using Tremolo as a spectral library search tool (Wang and Bandeira, 2013). The parameters used to proceed to the benchmarking dataset analysis were the following: parent mass tolerance 0.05 Da, minimum cosine score 0.1, no limits for the number of returned candidates.

#### MS-Finder

This *in silico* fragmentation approach considers multiple parameters such as bond dissociation energies, mass accuracies, fragment linkages and various hydrogen rearrangement rules at the candidate ranking phase (Tsugawa et al., 2016). The resulting scoring system range from 1 to 10. The parameters used to proceed to the benchmarking dataset analysis were the following: mass tolerance setting: 0.1 Da (MS1), 0.1 Da (MS2); relative abundance cut off: 5% formula finder settings: LEWIS and SENIOR check (yes), isotopic ratio tolerance: 20%, element probability check (yes), element selection (O, N, P, S, Cl, Br). Structure Finder setting: tree depth: 2, maximum reported number: 100, data sources (all except MINEs DBs. Total number of structures, 321,617.) MS-Finder v. 3.22 was used, it is available at the following address: http://prime.psc.riken.jp/Metabolomics_Software/MS-FINDER/. 

#### Sirius

Sirius 4.0.1 is considered as a state-of-the-art metabolite annotation solution, which combines molecular formula calculation and the prediction of a molecular fingerprint of a query compound from its fragmentation tree and spectrum (Dührkop et al., 2019). Sirius uses a DB of 73,444,774 unique structures for its annotations. The parameters used to proceed to the benchmarking dataset analysis were the following for Sirius molecular formula calculation: possible ionization [M+H]^+^, instrument: Q-TOF, ppm tolerance 50 ppm, Top molecular formula candidates: 3, filter:formulas from biological DBs. For the CSI : FingerID step, the parameters were the following: possible adducts: [M+H]^+^, filter: compounds present in biological DB, maximal number of returned candidates: unlimited. Sirius 4.0.1 is available at the following address: https://bio.informatik.uni-jena.de/software/sirius/.

### Results Analysis

The F1 score was calculated for each evaluated metabolite annotation tool before and after the *taxonomically informed scoring* step. The F1 score is the harmonic mean of the recall (True Positive/(True Positive + False Negative)) and precision rate (True Positive/(True Positive + False Positive)) of a tool. The True Positive (TP) corresponds to the number of correct candidate annotations at rank 1, the False Positive (FP) to the number of wrong candidate annotations at rank 1, and the False Negative (FN) to the number of correct annotations at rank >1. The F1 score is then calculated as follows:

$$F1\;score\;=\;2\;\times \;\frac{\left(Recall\;rate\times Precision\;rate\right)}{\left(Recall\;rate+Precision\;rate\right)}$$

An R notebook to analyze the results of the *taxonomically informed scoring* process and plot the figures of this manuscript is available online (taxo_figures.Rmd) at https://github.com/oolonek/taxo_scorer. 

### Optimization of the Scores Combination for the Taxonomically Informed Scoring

In order to establish the optimal scores to be applied for each of the taxonomic distances (family, genus and species), the information related to candidate annotations was artificially degraded. For this, the annotation dataset returned by the ISDB-DNP approach against the benchmarking dataset was randomized. The randomized annotation dataset was then split into four equal blocks. For the first three blocks, the biological source information was deleted, respectively, at the species level; at the genus and species level; and, finally, at the family, genus and species levels. The fourth block was not modified. Finally, the four blocks were merged back to a unique dataset. The process was repeated four times yielding four datasets with randomly incomplete biological sources. The taxonomic distance informed scoring process was compiled to a unique function taking three arguments (scores given when a match was found at the family, genus and species level, respectively) and outputting the number of correct hits ranked at the first position. A parallelizable Bayesian optimization algorithm (https://github.com/AnotherSamWilson/ParBayesianOptimization) was then used, being particularly suited for the optimization of black box functions for which no formal representation is available (arXiv:1807.02811). The bounds were set between 0 and 3 for the exploration of the three parameters of the function. Number of initial points was set to 10 and the number of iterations to 100. Parameter kappa (κ) was set to 5.152, to force the algorithm to explore unknown areas. The chosen acquisition function was set to Expected Improvement (ei). Epsilon parameter (ε, eps) was set to 0. The whole procedure was run 4 times on the 4 randomized datasets. Best set of parameters were then averaged across the 16 results set. All codes required for this optimization step are available online (3_taxo_optimizer.Rmd) at https://github.com/oolonek/taxo_scorer. 

### Chemical Analysis and Isolation of Compounds From Glaucium Extract

#### Plant Material

The aerial flowering parts of three *Glaucium* species were collected in May and June of 2015 from the northern part of Iran including Mazandaran and Tehran provinces. The samples were identified by Dr. Ali Sonboli, Medicinal Plants and Drugs Research Institute, Shahid Beheshti University, Tehran, Iran. The voucher specimens MPH-2351 for *G. grandiflorum* (vernacular Shaghayegh goldrosht), MPH-2352 for *G. fimbrilligerum* (vernacular Shaghayegh sharabeie) and MPH-2353 for *G. corniculatum* (vernacular Shaghayegh shakhdar or red horned poppy) have been deposited at the Herbarium of Medicinal Plants and Drugs Research Institute (HMPDRI), Shahid Beheshti University, Tehran, Iran.

#### Mass Spectrometry Analysis

Chromatographic separation was performed on a Waters Acquity UPLC system interfaced to a Q-Exactive Focus mass spectrometer (Thermo Scientific, Bremen, Germany), using a heated electrospray ionization (HESI-II) source. Thermo Scientific Xcalibur 3.1 software was used for instrument control. The LC conditions were as follows: column, Waters BEH C18 50 × 2.1 mm, 1.7 μm; mobile phase, (A) water with 0.1% formic acid; (B) acetonitrile with 0.1% formic acid; flow rate, 600 μl·min^−1^; injection volume, 6 μl; gradient, linear gradient of 5−100% B over 7 min and isocratic at 100% B for 1 min. The optimized HESI-II parameters were as follows: source voltage, 3.5 kV (pos); sheath gas flow rate (N_2_), 55 units; auxiliary gas flow rate, 15 units; spare gas flow rate, 3.0; capillary temperature, 350.00°C, S-Lens RF Level, 45. The mass analyzer was calibrated using a mixture of caffeine, methionine–arginine–phenylalanine–alanine–acetate (MRFA), sodium dodecyl sulfate, sodium taurocholate, and Ultramark 1621 in an acetonitrile/methanol/water solution containing 1% formic acid by direct injection. The data-dependent MS/MS events were performed on the three most intense ions detected in full scan MS (Top3 experiment). The MS/MS isolation window width was 1 Da, and the stepped normalized collision energy (NCE) was set to 15, 30 and 45 units. In data-dependent MS/MS experiments, full scans were acquired at a resolution of 35,000 FWHM (at *m/z* 200) and MS/MS scans at 17,500 FWHM both with an automatically determined maximum injection time. After being acquired in a MS/MS scan, parent ions were placed in a dynamic exclusion list for 2.0 s.

#### MS Data Pretreatment

The MS data were converted from .RAW (Thermo) standard data format to .mzXML format using the MSConvert software, part of the ProteoWizard package (Chambers et al., 2012). The converted files were treated using the MZMine software suite v. 2.38 (Pluskal et al., 2010).

The parameters were adjusted as following: the centroid mass detector was used for mass detection with the noise level set to 1.0E6 for MS level set to 1, and to 0 for MS level set to 2. The ADAP chromatogram builder was used and set to a minimum group size of scans of 5, minimum group intensity threshold of 1.0E5, minimum highest intensity of 1.0E5 and *m/z* tolerance of 8.0 ppm. For chromatogram deconvolution, the algorithm used was the wavelets (ADAP). The intensity window S/N was used as S/N estimator with a signal to noise ratio set at 25, a minimum feature height at 10,000, a coefficient area threshold at 100, a peak duration ranges from 0.02 to 0.9 min and the RT wavelet range from 0.02 to 0.05 min. Isotopes were detected using the isotopes peaks grouper with a *m/z* tolerance of 5.0 ppm, a RT tolerance of 0.02 min (absolute), the maximum charge set at 2 and the representative isotope used was the most intense. An adduct (Na^+^, K^+^, NH_4_^+^, CH_3_CN^+^, CH_3_OH^+^, C_3_H_8_O^+^ (IPA^+^)) search was performed with the RT tolerance set at 0.1 min and the maximum relative peak height at 500%. A complex search was also performed using [M+H]^+^ for ESI positive mode, with the RT tolerance set at 0.1 min and the maximum relative peak height at 500%. Peak alignment was performed using the join aligner method (*m/z* tolerance at 8 ppm), absolute RT tolerance 0.065 min, weight for *m/z* at 10 and weight for RT at 10. The peak list was gap-filled with the same RT and *m/z* range gap filler (*m/z* tolerance at 8 ppm). Eventually the resulting aligned peaklist was filtered using the peak-list rows filter option in order to keep only features associated with MS2 scans.

#### Molecular Networks Generation

In order to keep the retention time, the exact mass information and to allow for the separation of isomers, a feature based molecular network (https://ccms-ucsd.github.io/GNPSDocumentation/featurebasedmolecularnetworking/) was created using the .mgf file resulting from the MZMine pretreatment step detailed above. Spectral data was uploaded on the GNPS molecular networking platform. A network was then created where edges were filtered to have a cosine score above 0.7 and more than six matched peaks. Further edges between two nodes were kept in the network if and only if each of the nodes appeared in each other’s respective top 10 most similar nodes. The spectra in the network were then searched against GNPS’ spectral libraries. All matches kept between network spectra and library spectra were required to have a score above 0.7 and at least six matched peaks. The output was visualised using Cytoscape 3.6 software (Shannon et al., 2003). The GNPS job parameters and resulting data are available at the following address (https://gnps.ucsd.edu/ProteoSAFe/status.jsp?task=a475a78d9ae8484b904bcad7a16abd1f). 

#### Taxonomically Informed Metabolite Annotation

The spectral file (.mgf) and attributes metadata (.clustersummary) obtained after the MN step were annotated using the ISDB-DNP with the following parameters: parent mass tolerance 0.005 Da, minimum cosine score 0.2, maximal number of returned candidates: 50. An R script was written to proceed to the *taxonomically informed scoring* on GNPS outputs and return an attribute table which can be directly loaded in Cytoscape. The script is available online (taxo_scorer_user.Rmd) at https://github.com/oolonek/taxo_scorer. 

#### Isolation of Predicentrine and Glaucine From *G. grandiflorum*

The air-dried, ground and powdered plant materials (500 g) was successively extracted by solvents of increasing polarities (hexane, ethyl acetate and methanol), 4 × 5.0 L of each solvent (48 h). An aliquot of each ethyl acetate and methanolic extract was submitted to C18 SPE (eluted with 100% MeOH), dried under nitrogen flow and redissolved at 5 mg/ml in MeOH for LC–MS analysis. The methanolic extract of *G. grandiflorum* was concentrated under reduced pressure, then dried with a nitrogen flow until complete evaporation of the residual solvent yielding 50 g of extract. An aliquot (5 g) was subjected to a VLC in order to eliminate sugars and other very polar compounds. A 250 ml sintered-glass Buchner funnel connected to a vacuum line was packed with a C18 reverse phase LiChroprep 40–63 μm (Lobar Merck, Darmstadt, Germany). After conditioning the stationary phase with methanol (4 × 250 ml, 0.1% formic acid) and distilled water (4 × 250 ml, 0.1% formic acid), 5 g of methanolic extract was dissolved in water and the mixture was deposited on the stationary phase. Elution of the sample was conducted using water (4 × 250 ml, 0.1% formic acid) in the first step and followed by methanol (4 × 250 ml, 0.1% formic acid) in the second step. This process yielded 1.4 g of processed methanolic extract. After condition optimisation at the analytical level, 50 mg of the extract were solubilized in 500 µl DMSO and injected using a Rheodyne^®^ valve (1 ml loop). Semi-preparative HPLC-UV purification was performed on a Shimadzu system equipped with: LC20A module elution pumps, an SPD-20A UV/VIS detector, a 7725I Rheodyne^®^ injection valve, and a FRC-10A fraction collector (Shimadzu, Kyoto, Japan). The HPLC system was controlled by the LabSolutions software. The HPLC conditions were selected as follows: Waters X-Bridge C18 column (250 × 19 mm i.d., 5 μm) equipped with a Waters C18 pre-column cartridge holder (10 × 19 mm i.d.); solvent system consists of ACN (2 mM TEA) (B) and H_2_O (2 mM TEA & 2 mM ammonium acetate) (A). Optimized separation condition from the analytical was transferred to semi-preparative scale by a geometric gradient transfer software (Guillarme et al., 2008). The separation was conducted in gradient elution mode as follows: 5% B in 0–5 min, 12% B in 5–10 min, 30% B in 10–30 min, 60% B in 30–55 min, 100% B in 55–65 min. The column was reconditioned by equilibration with 5% of B in 15 min. Flow rate was equal to 17 ml/min and UV traces were recorded at 210 nm and 280 nm. The separation procedure yielded 0.3 mg of predicentrine and 3.4 mg of glaucine. Spectra for predicentrine (CCMSLIB00005436122) and glaucine (CCMSLIB00005436123) were deposited on GNPS servers.

#### NMR Analysis

The NMR spectra of each isolated compound was recorded on a Bruker BioSpin 600 MHz spectrometer (Avance Neo 600). Chemical shifts (δ) were recorded in parts per million in methanol‒d4 with TMS as an internal standard. NMR data are available as **Supplementary Material S1** and **S2**.

## Data Availability Statement

Scripts and datasets generated and analyzed for this study can be found on github (scripts): https://github.com/oolonek/taxo_scorer and at the following OSF repository (datasets): https://osf.io/bvs6x/(DOI 10.17605/OSF.IO/BVS6X). This manuscript has been released as a pre-print at bioRxiv: http://dx.doi.org/10.1101/702308. 

## Author Contributions

P-MA, AR, MD-K, and J-LW designed the study. JB wrote the Python script for gnfinder output formatting. P-MA and AR wrote the scripts for dataset preparation, *taxonomically informed scoring*, and results analysis. P-MA, AR, MD-K, SO, MB, and TS used the scripts for metabolite annotation and provided feedback. MB and SNE collected the *Glaucium* species. P-MA and AR performed the LCMS analysis on the *Glaucium* extracts. MB analyzed profiling data, isolated the compounds of *Glaucium*, and established their structures. P-MA wrote the manuscript together with AR and J-LW. All authors discussed the results and commented on the manuscript.

## Funding

JB gratefully acknowledges the support of this work by grant U41 AT008706 from NCCIH and ODS. J-LW is thankful to the Swiss National Science Foundation for the support in the acquisition of the NMR 600 MHz (SNF R’Equip grant 316030_164095).

## Conflict of Interest

The authors declare that the research was conducted in the absence of any commercial or financial relationships that could be construed as a potential conflict of interest.
