# Supplementary material for: Taxonomically Informed Scoring Enhances Confidence in Natural Products Annotation
Source: Front Plant Sci. 2019 Oct 25;10:1329. doi: 10.3389/fpls.2019.01329 (PMC6824209; doi:10.3389/fpls.2019.01329)
Supplement: Supplementary file 2 [file DataSheet_2.pdf]

Supplementary Material

Taxonomically informed scoring enhances confidence in natural products annotation

The following Supplementary Material is available for this article:

S1. Structural elucidation of predicentrine 2
S2. Structural elucidation of glaucine 6
S3. Overview of the benchmarking dataset establishment 10
S4. ROC curves (number of correct annotations vs. rank). Full view and zoom 11
S5. Output of the taxonomically informed scoring annotation using ISDB-DNP for feature m/z 356.1860 at 1.83 min. 12
S6. Cluster related to predicentrine in *Glaucium* extract..13
S7. Mosaic plot of the instrument sources vs. library origin vs number of entries in the benchmarking set..14

S1. Structural elucidation of predicentrine

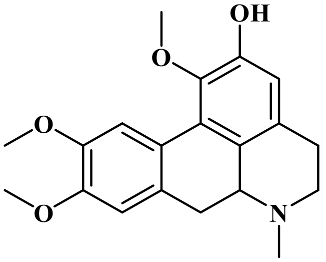

Predicentrine: ESI-HRMS (*m/z*): 342.1699 $[M + H]^+$; ^{1}H NMR (**600 MHz,Methanol-*d₃***) δ: 2.55 (1H, obsc, H-7b), 2.62 (1H,obsc , H-5b), 2.56 (3H, s, N–CH3), 2.70 (1H, dd, *J*=15.6, 4 Hz,H-4b), 3.12 (1H, obsc, H-6a), 3.14(1H, obsc, H-5a), 3.15 (1H, obsc, H-7a), 3.1 (1H, obsc, H-4a), 3.6 (3H, s, O–CH3), 3.88 (3H, s, O–CH3), 3.90 (3H, s, O–CH3), 6.6 (1H, s,H-3), 6.94 (1H, s,H-8), 8.03(1H, s,H-11), ^{13}C NMR (**600 MHz,Methanol-*d₃***), δ: 26.98 (C-4), 32.77(C-7), 41.62 (N-CH3), 52.06 (C-5), 54.22 (C_9-OCH_3), 54.45 (C_{10}-OCH_3), 58.31 (C_1-OCH_3), 148.78 (C-2), 61.87 (C-6a), 113.4(C-3), 110.56 (C-8), 110.98 (C-11), 123.67 (C-11a), 124.06 (C-6b), 125.58(C-11b), 127.74 (C-3a), 128.30 (C-7a), 142.50 (C-1), 147.2 (C-10), 147.50 (C-9). **(* Not assigned with certainty. "obsc.": The signal is obscured by overlapping peaks)**

MSMS spectrum of predicentrine was deposited on the GNPS librairies (CCMSLIB00005436122).

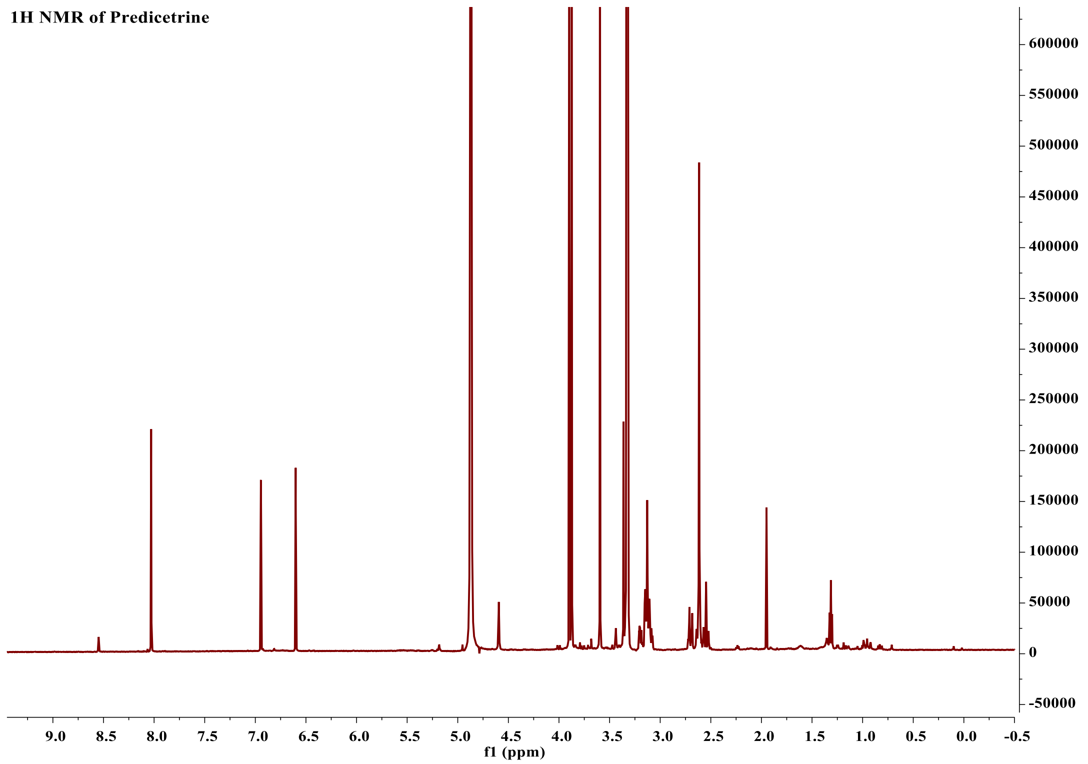

Figure 1 1H NMR NMR spectrum of predicentrine

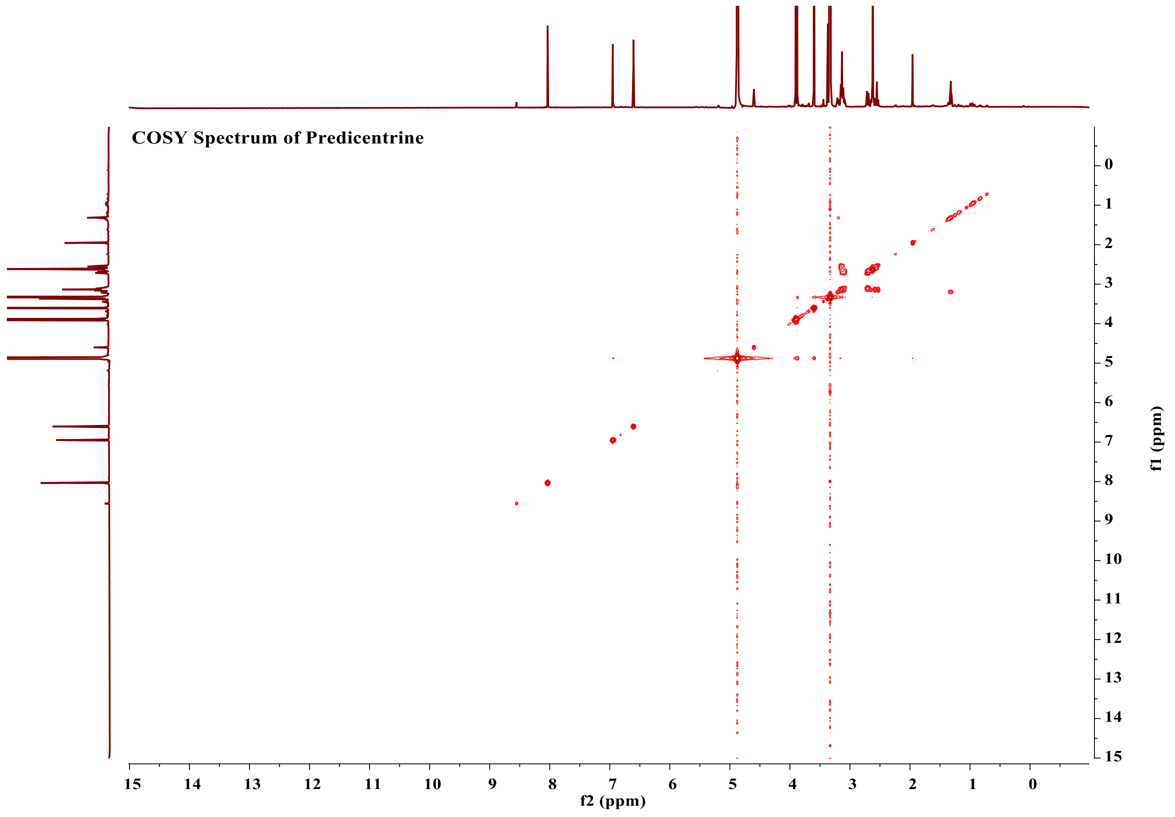

Figure 2 COSY spectrum of predicentrine

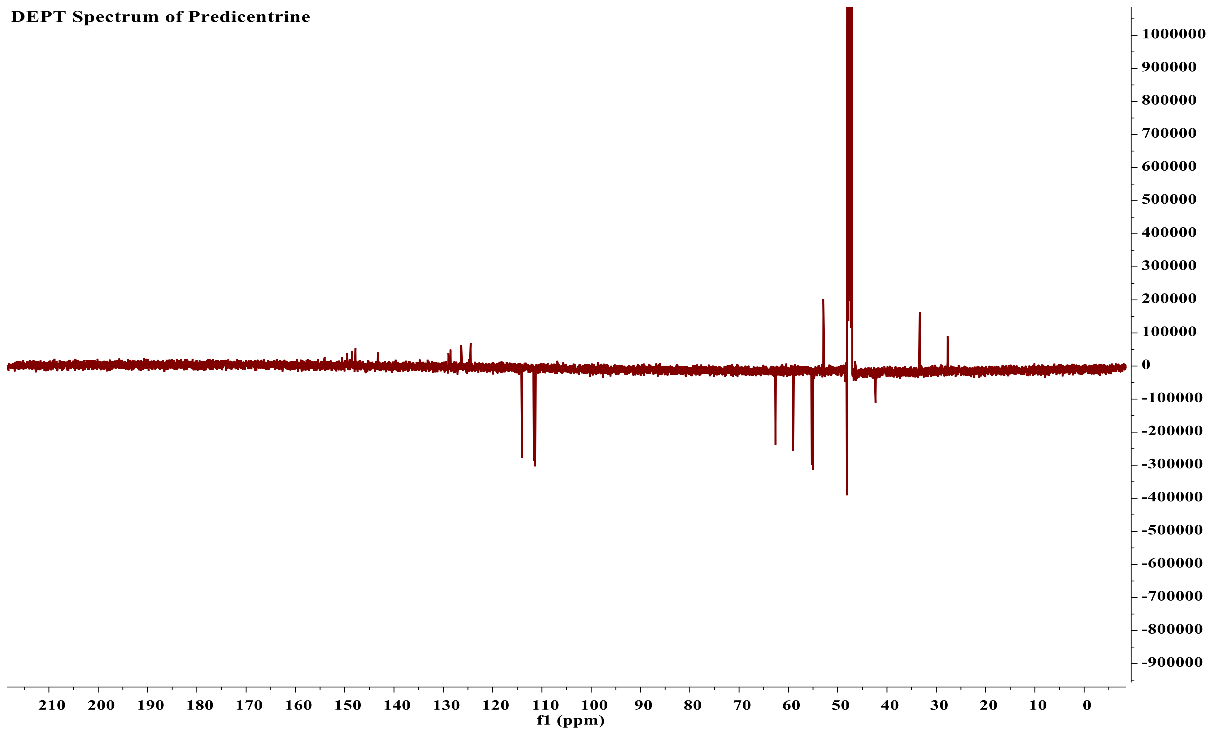

Figure 3 DEPT spectrum of predicentrine

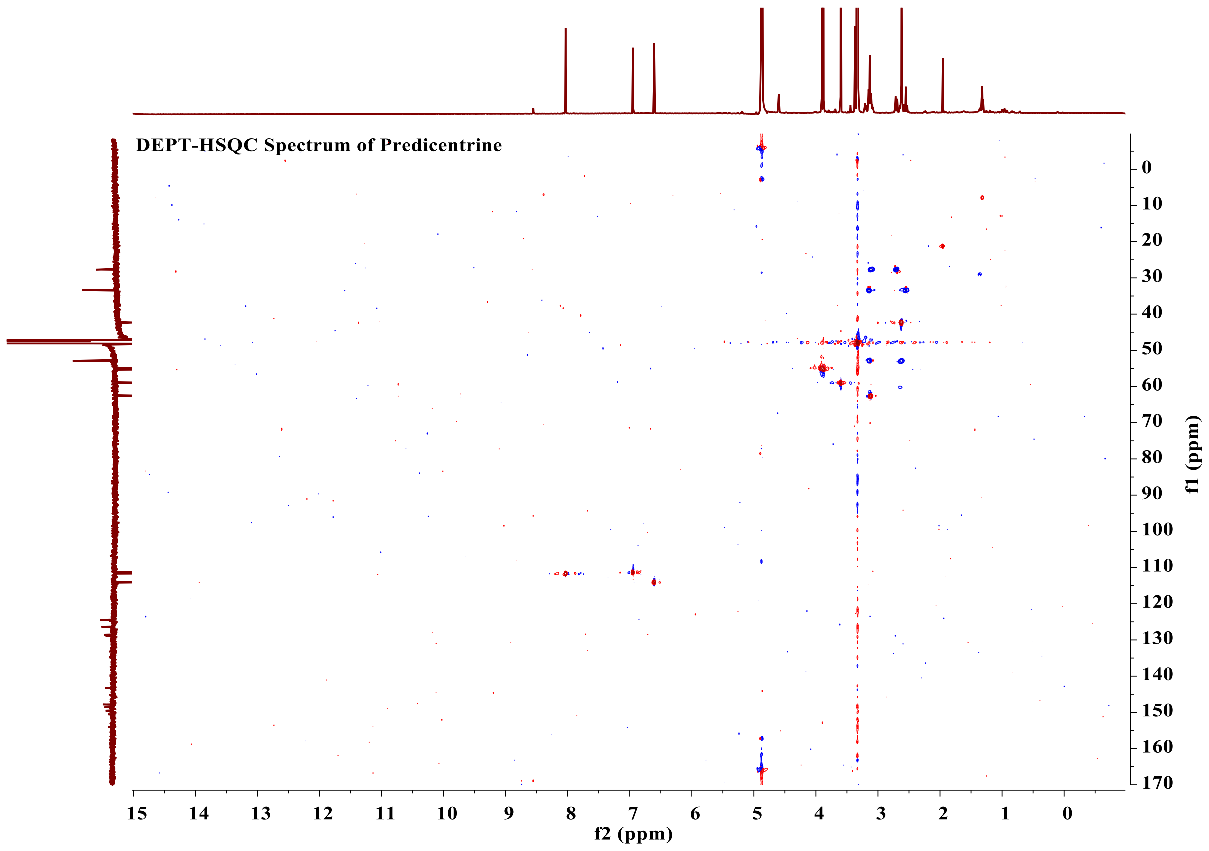

Figure 4 DEPT-HSQC spectrum of predicentrine

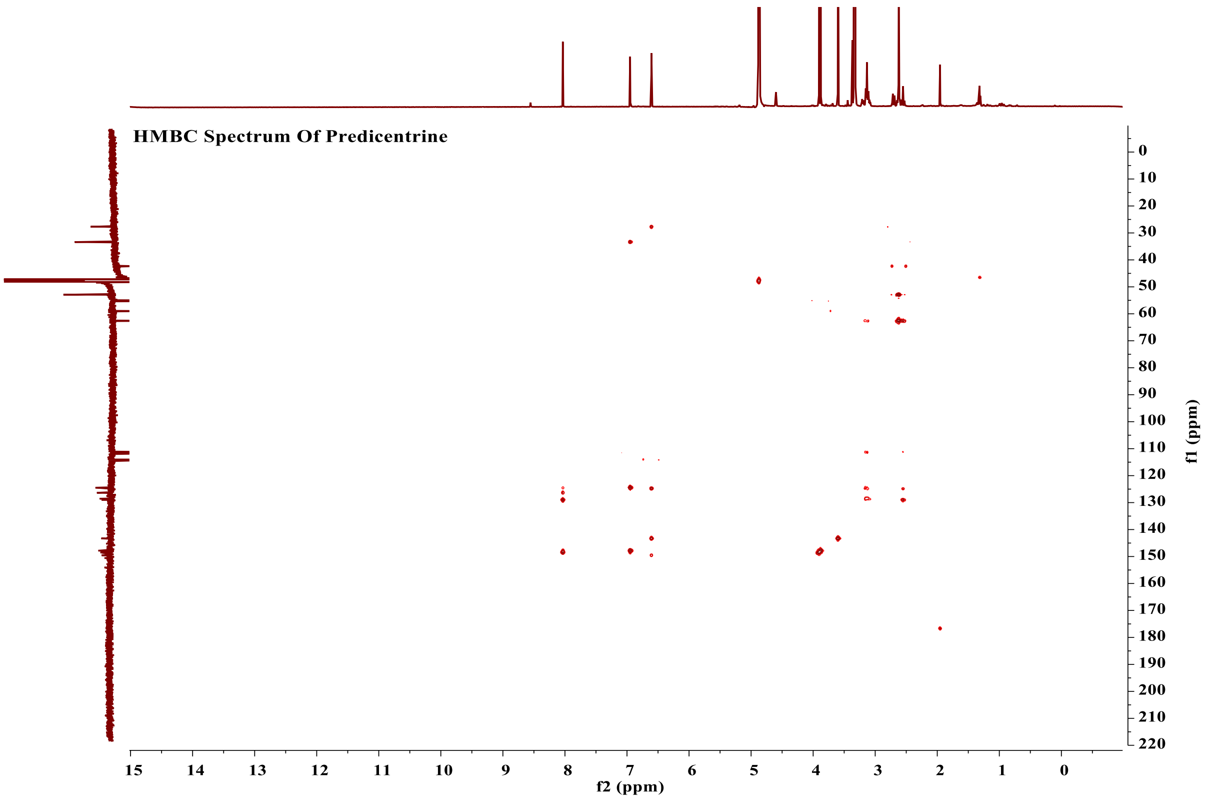

Figure 5 HMBC spectrum of predicentrine

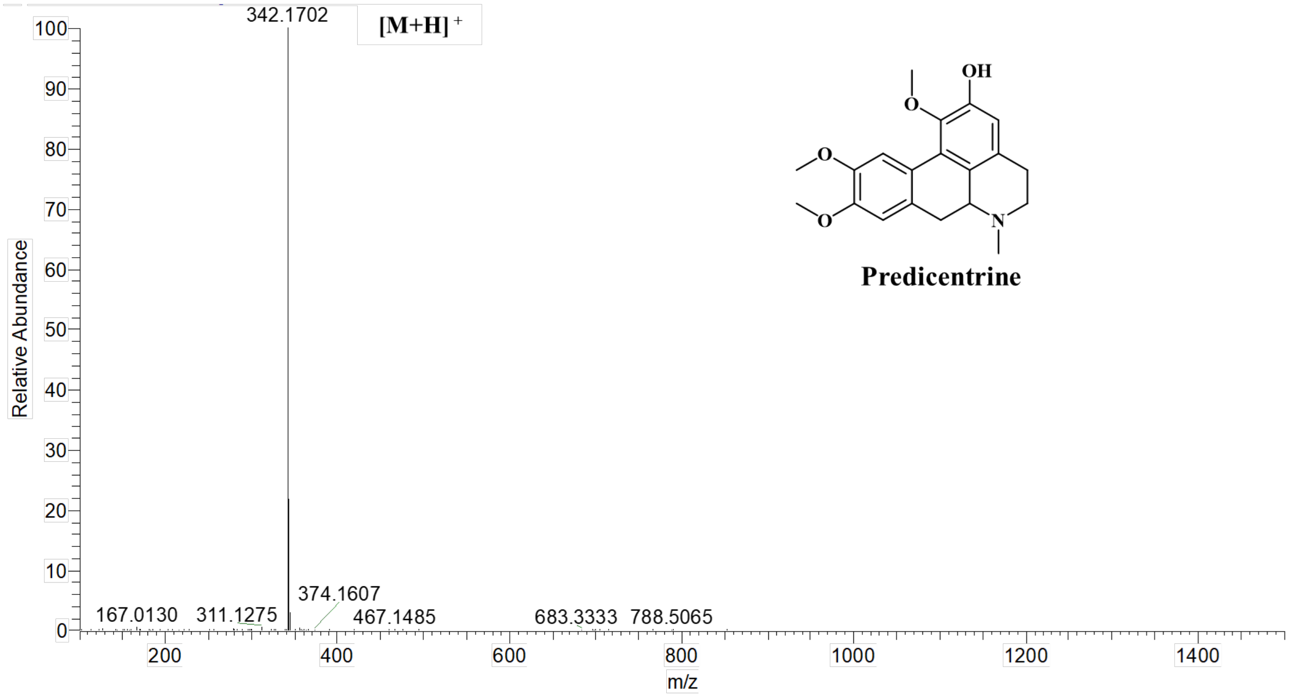

Figure 6 HRMS spectrum of predicentrine

S2. Structural elucidation of glaucine

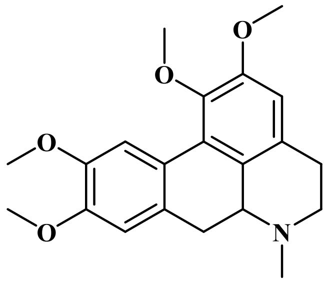

Glaucine: ESI-HRMS (*m/z*): 356.1859 $[M + H]^+$; ^{1}H NMR (**600 MHz,Methanol-*d₃***) δ: 2.51 (1H, obsc, H-7b), 2.57 (1H, m, H-5b), 2.59 (3H, s, N–CH3), 2.75 (1H, dd, *J*=15.5, 3.7 Hz,H-4b), 3.07 (1H, dd, *J*=13.7, 3.9 Hz), 3.11(1H, obsc, H-5a), 3.14 (1H, obsc, H-7a), 3.15 (1H, obsc, H-4a), 3.64 (3H, s, O–CH3), 3.87 (3H, s, O–CH3), 3.88 (3H, s, O–CH3), 3.89 (3H, s, O–CH3), 6.74 (1H, s,H-3), 6.93 (1H, s,H-8), 8.01(1H, s,H-11), ^{13}C NMR (**600 MHz,Methanol-d_3**), δ: 27.47 (C-4), 32.77(C-7), 41.79 (N-CH3), 52.17(C-5), 54.35 (C_9-OCH_3), 54.35 (C_{10}-OCH_3), 54.31 (C_1-OCH_3), 54.35 (C_2-OCH_3), 61.72 (C-6a), 109.55(C-3), 110.57 (C-8), 111.34 (C-11), 123.81 (C-11a), 151.68 (C-2), 125.36 (C-6b), 125.83(C-11b), 127.74 (C-3a), 128.55 (C-7a), 143.69(C-1), 146.83 (C-10), 147.81 (C-9). **(* Not assigned with certainty. "obsc.": The signal is obscured by overlapping peaks)**

MSMS spectrum of glaucine was deposited on the GNPS librairies (CCMSLIB00005436123).

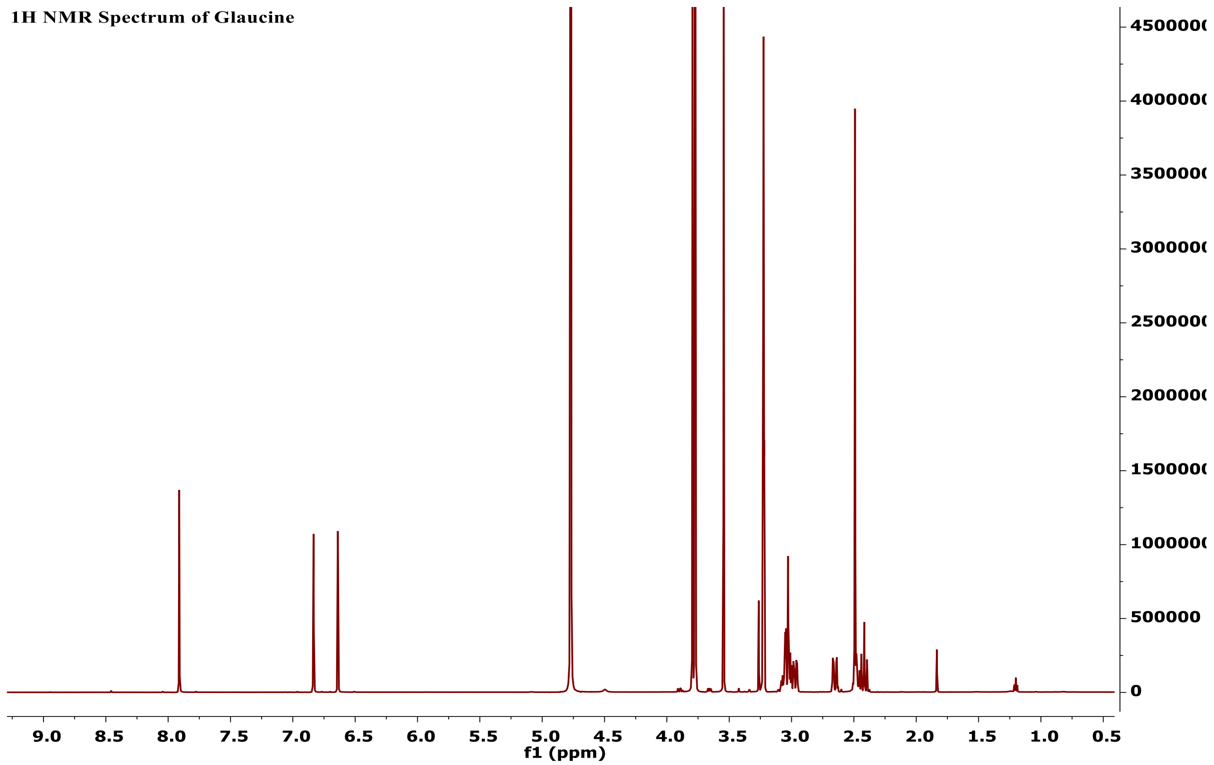

Figure 7 1HNMR NMR spectrum of glaucine

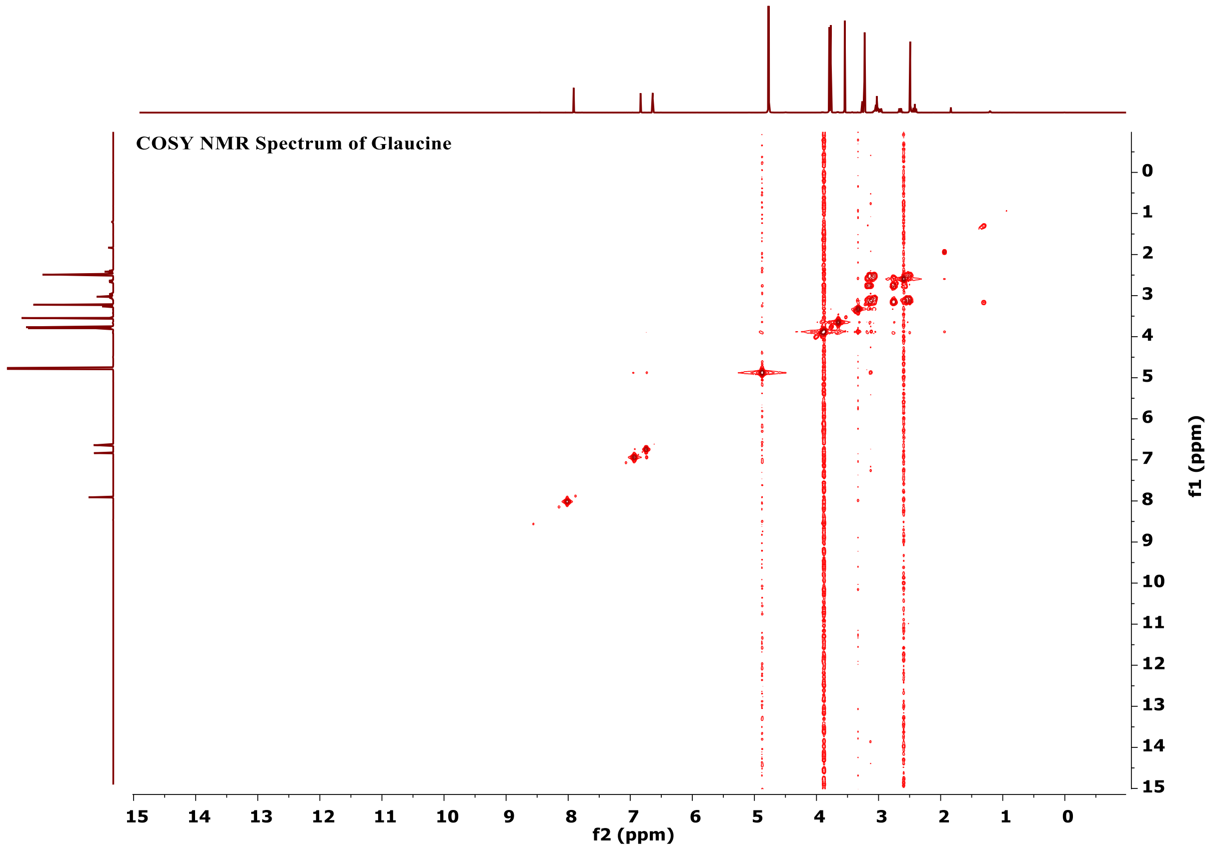

Figure 8 COSY NMR spectrum of glaucine

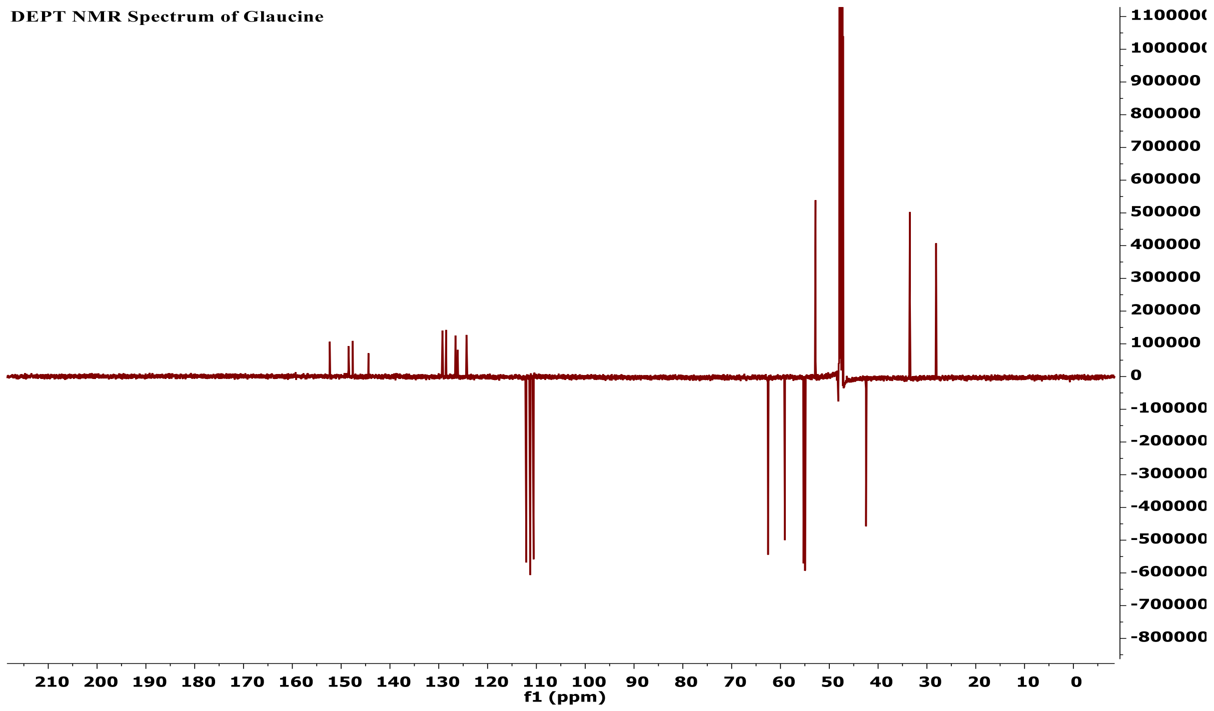

Figure 9 DEPT NMR spectrum of glaucine

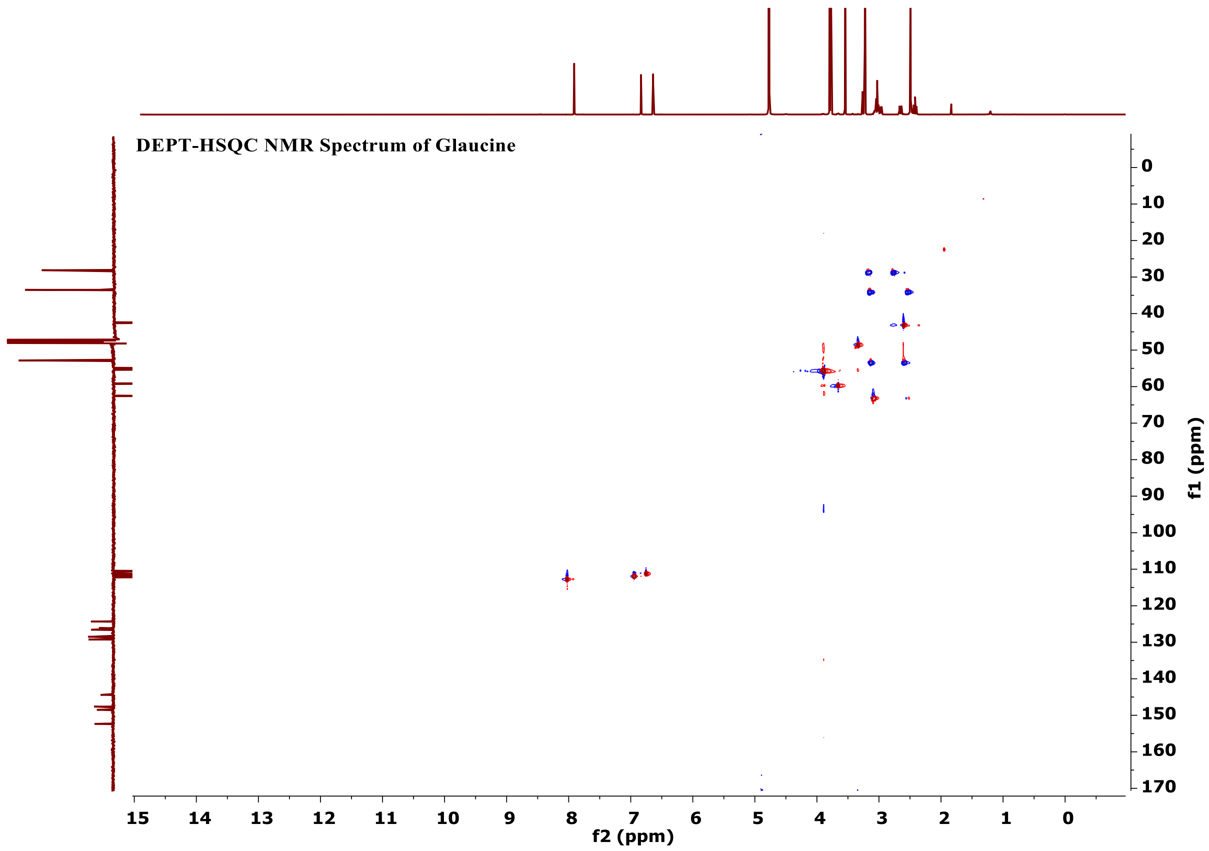

Figure 10 DEPT-HSQC NMR spectrum of glaucine

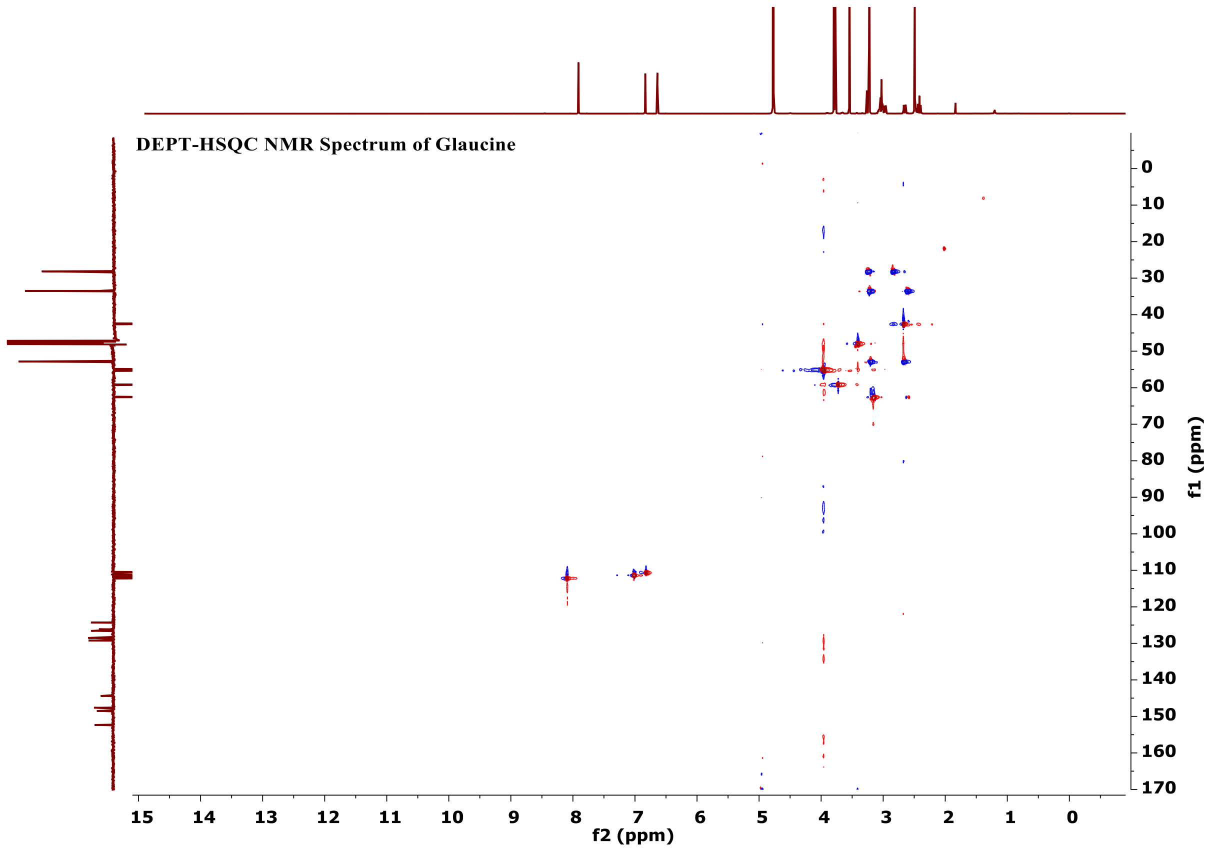

Figure 11 HMBC NMR spectrum of glaucine

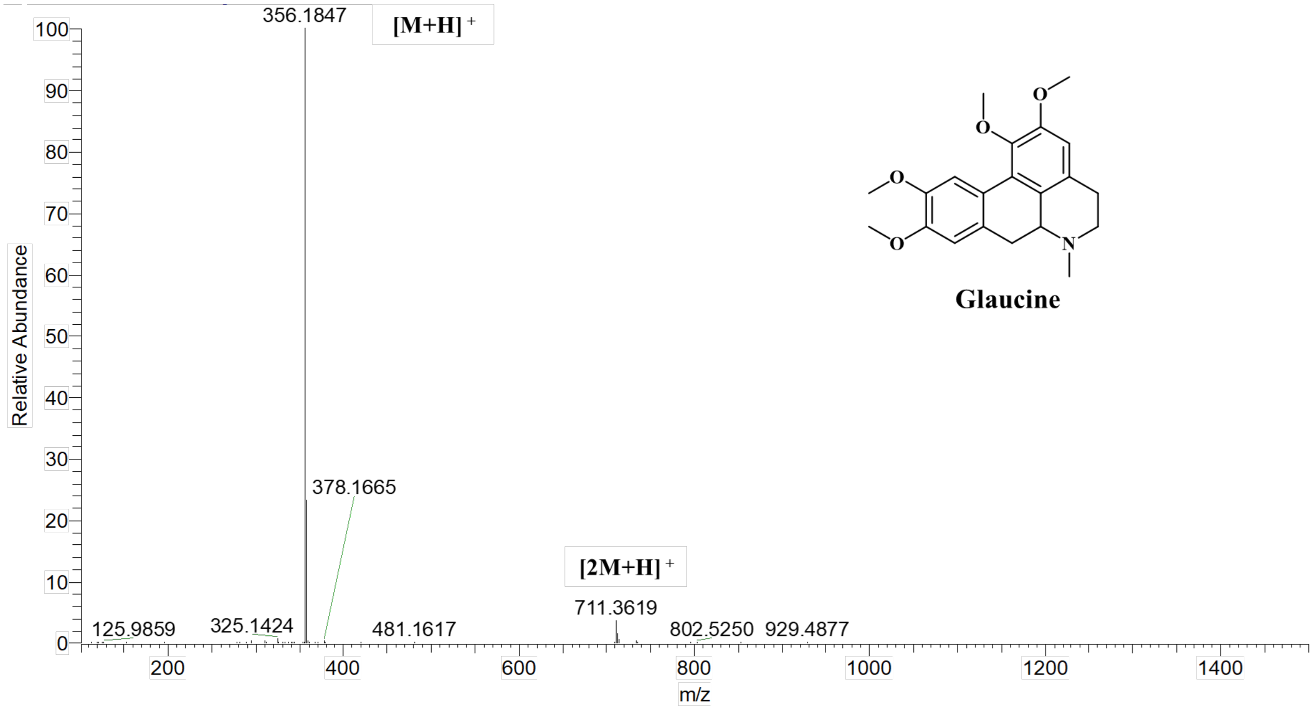

Figure 12 HRMS spectrum of glaucine

S3. Overview of the benchmarking dataset establishment

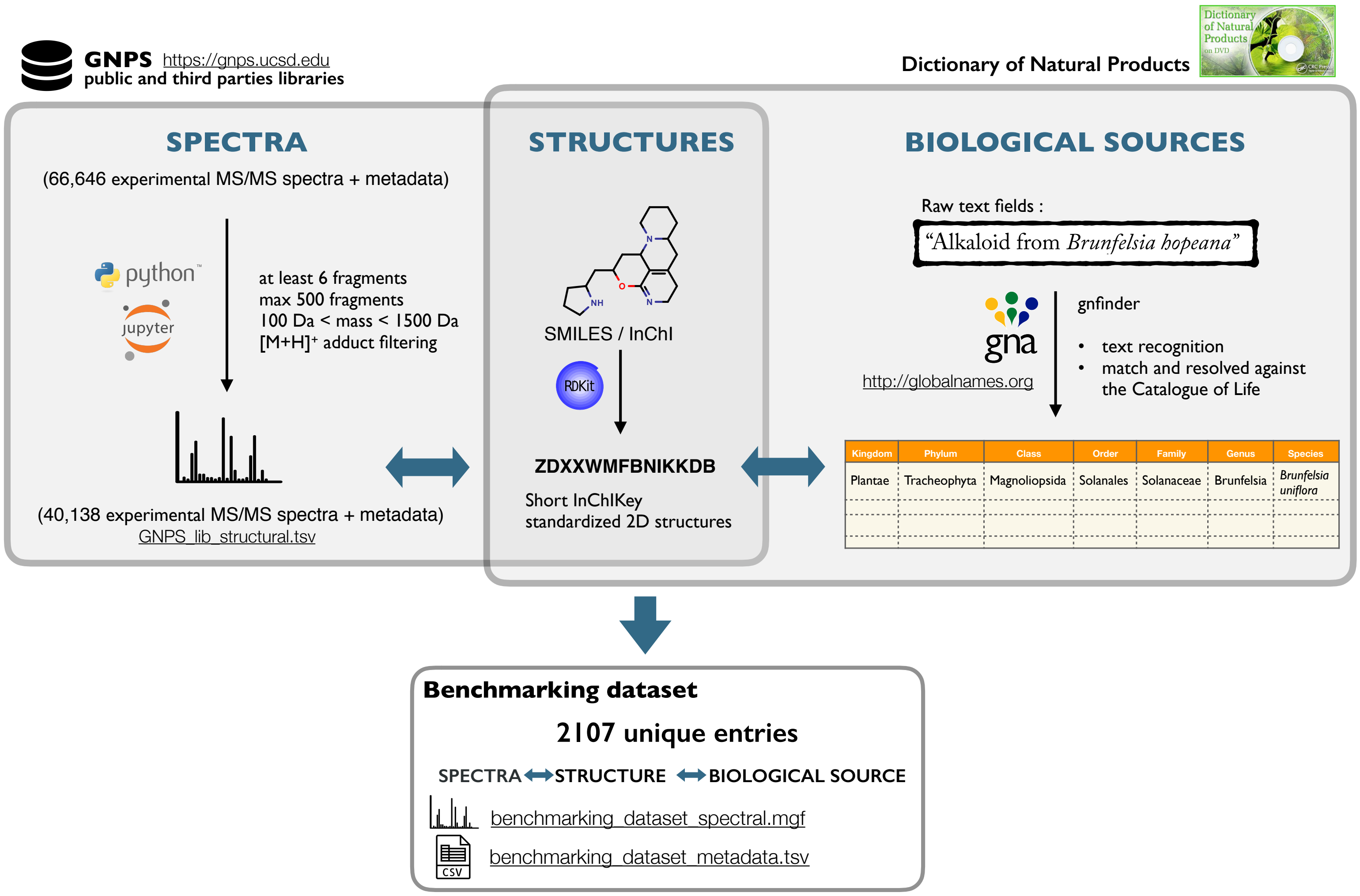

Figure 13 Overview of the benchmarking dataset establishment. The spectral/structural dataset is constituted from the GNPS libraries, the structural/biosource dataset is constituted from the DNP. NB: stereoisomers are not considered as datasets are linked based on common short InChIKey (standardized 2D structures). PDF version of this figure with clickable links is available at the following address: https://osf.io/bdg2j/

S4. ROC curves (number of correct annotations vs. rank). Full view and zoom

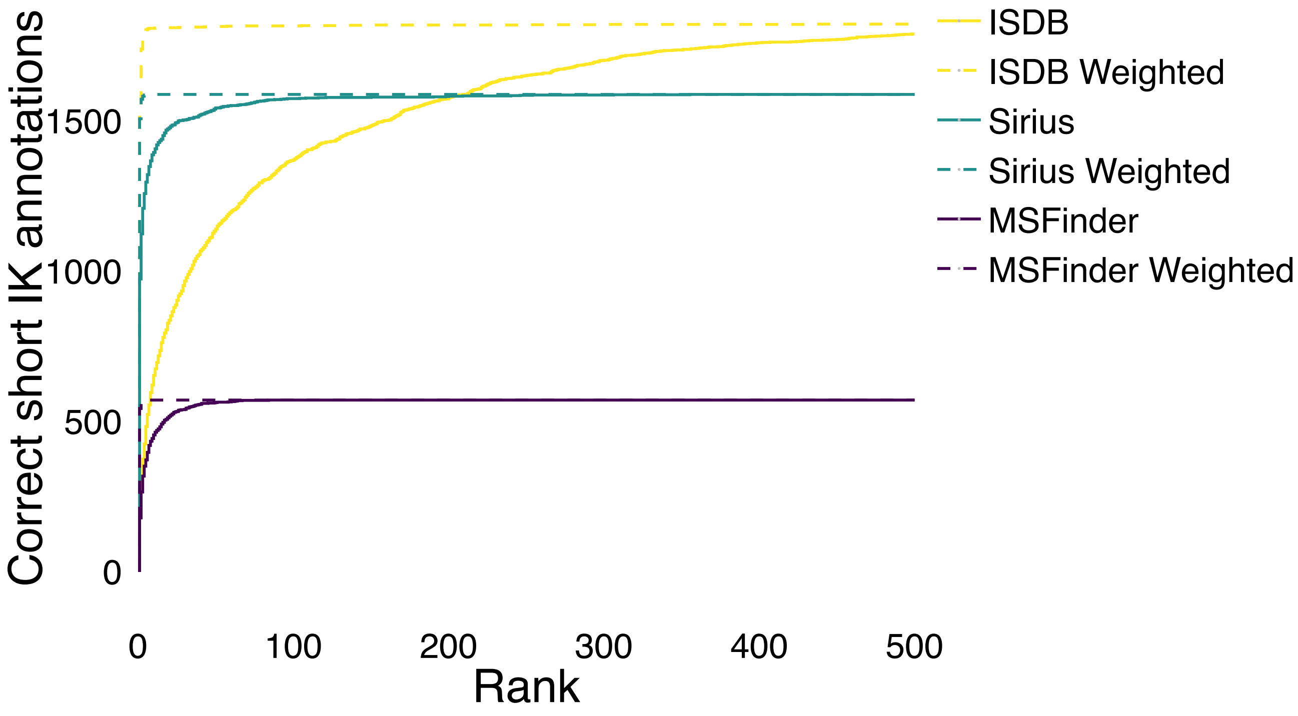

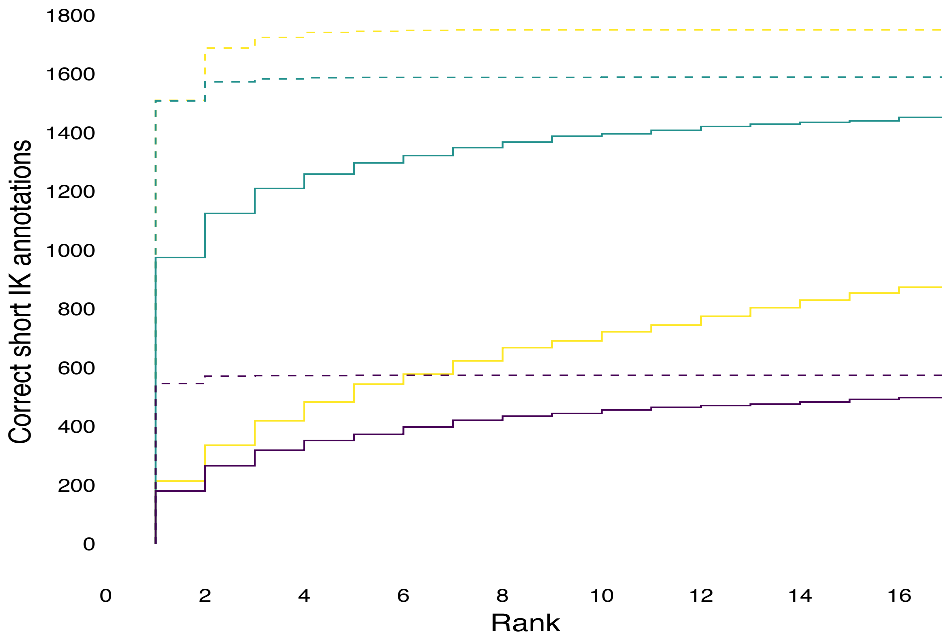

Cluster ID	Structure	Short IK	Molecule Name	Family	Genus	Species	Family Score	Genus Score	Species Score	Max Score	Spectral Score	Normalized Spectral Score	Combined Score	Rank Initial	Rank Final
**1771**	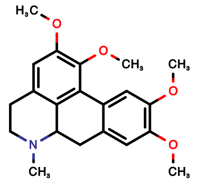	**RUZIU YOSRD WYQF**	**1,2,9,10-Tetrahydroxyaporphine Tetra-Me ether (Glaucine)**	**Papaveraceae**	***Glaucium***	**NA**	**0.81**	**1.62**	**0**	**1.62**	**0.43**	**0.36**	**1.98**	**7**	**1**
**1771**	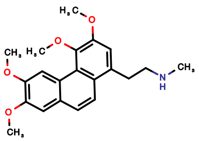	QGNLU OSBJAG YFF	N-Methylsecoglaucine N-De-Me	Papaveraceae	*Corydalis*	*Corydalis yanhusuo*	**0.81**	0	**0**	0.81	0.493	0.46	1.27	3	2
**1771**	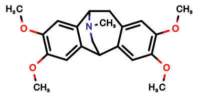	KUHFD ZAEYV THRS	Thalisopavine Me ether	Papaveraceae	*Papaver*	*Papaver radicatum*	**0.81**	0	**0**	0.81	0.455	0.4	1.21	4	3
**1771**	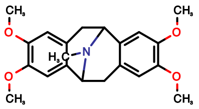	QEOWC PFWLCI QSL	Argemonine	Papaveraceae	*Argemone*	*Argemone gracilenta*	**0.81**	0	**0**	0.81	0.437	0.37	1.18	6	4
**1771**	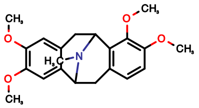	PSKQB NMDFP YFNM	Platycerine Me ether	Papaveraceae	*Argemone*	*Argemone platyceras*	**0.81**	0	**0**	0.81	0.428	0.35	1.17	8	5

S5. Output of the taxonomically informed scoring annotation using ISDB-DNP for feature m/z 356.1860 at 1.83 min. Glaucine, which is the correct annotation and was initially ranked at the 7th position, is now ranked at the first position.

S6. Cluster related to predicentrine in *Glaucium* extract.

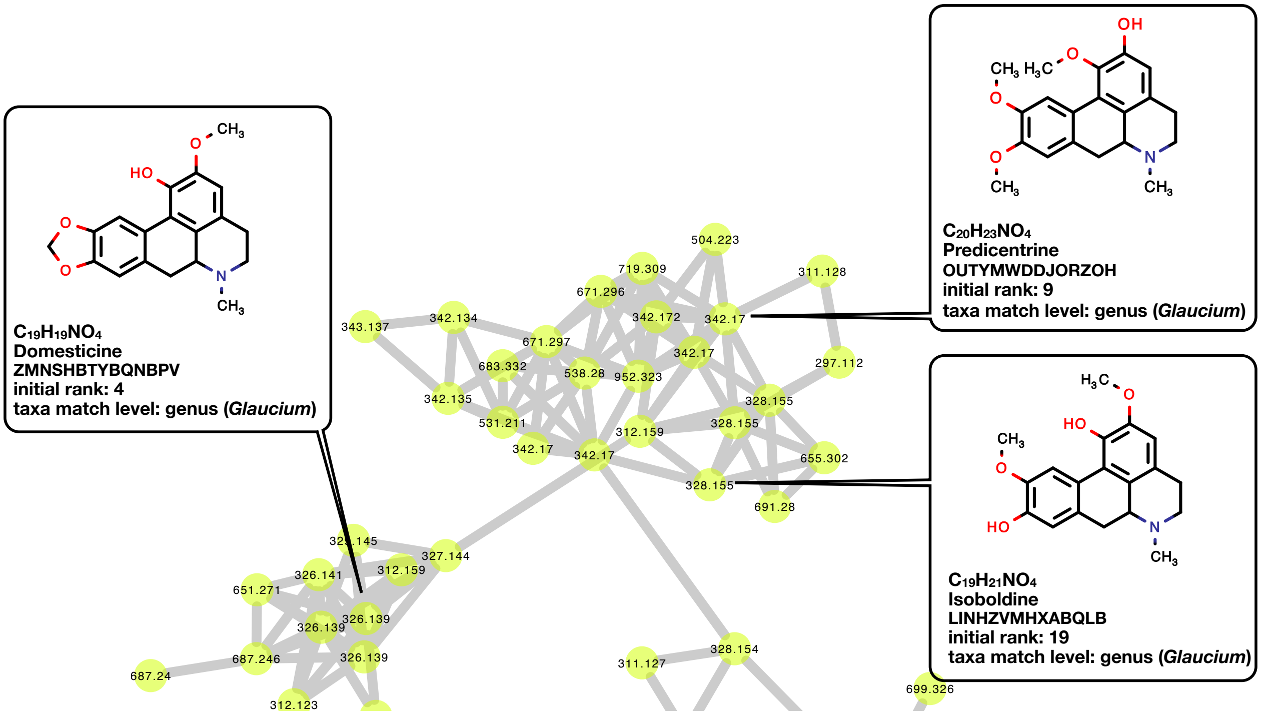

Figure 14 Depiction of additional annotations (rank 1) returned by the taxonomically informed scoring applied on the *Glaucium* extract. Predicentrine was isolated, other annotations are putative. Full MN is available online: (https://gnps.ucsd.edu/ProteoSAFe/status.jsp?task=a475a78d9ae8484b904bcad7a16abd1f)

S7. Mosaic plot of the instrument sources vs. library origin vs number of entries in the benchmarking set.

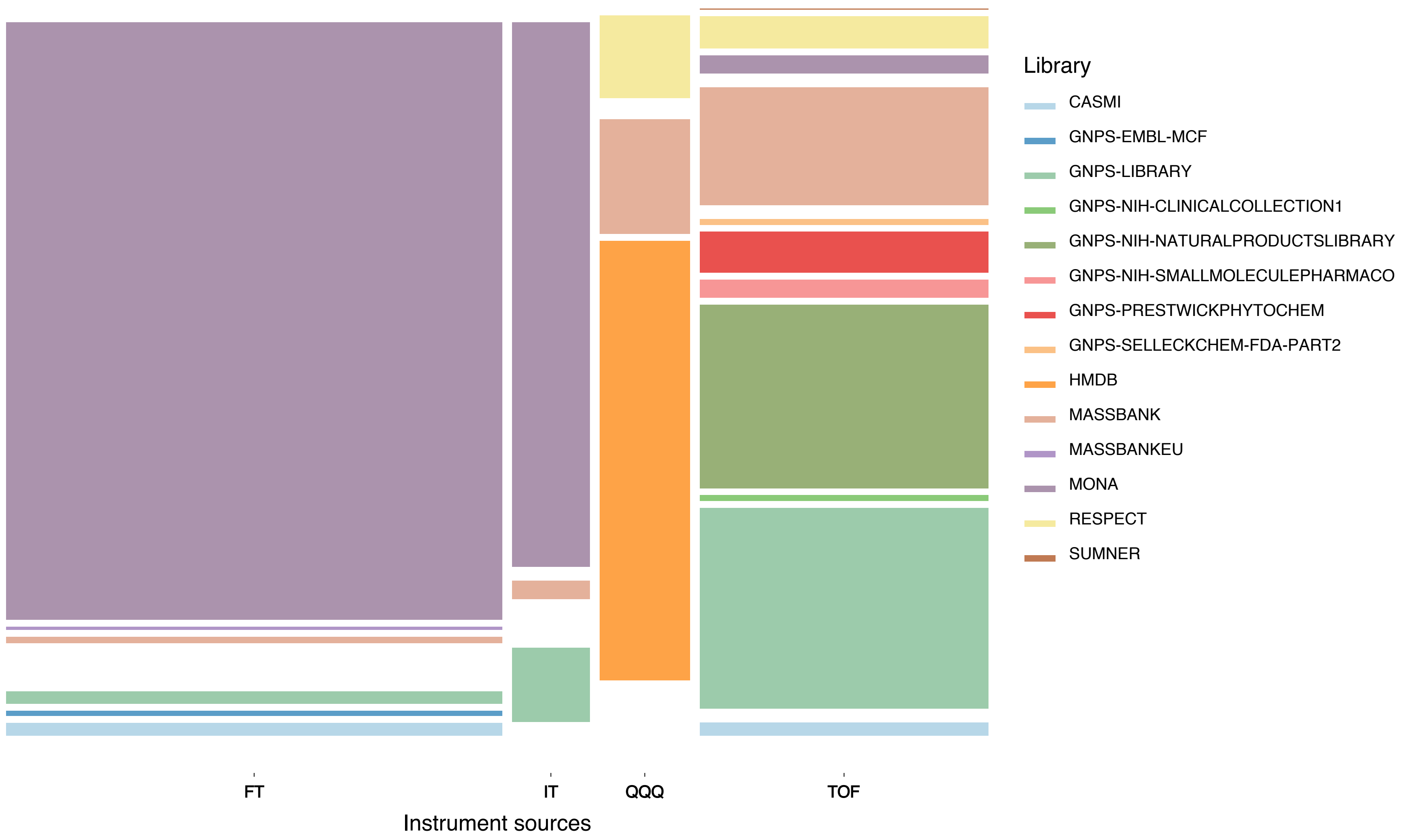

Figure 15 Mosaic plot of the instrument sources vs. library origin vs number of entries in the benchmarking set.
